# Biomarkers and Mechanisms of Male Infertility: Evaluation of Antioxidant Enzymes and Arachidonic Acid Derivatives in Seminal Plasma from Fertile and Infertile Men

**DOI:** 10.3390/antiox14121470

**Published:** 2025-12-07

**Authors:** Kamil Rodak, Izabela Kokot, Ricardo Faundez, Iwona Gilowska, Ewa Maria Kratz

**Affiliations:** 1 Division of Laboratory Diagnostics, Department of Laboratory Diagnostics, Faculty of Pharmacy, Wroclaw Medical University, Borowska Street 211A, 50-556 Wroclaw, Poland; izabela.kokot@umw.edu.pl; 2 Research and Development Center, INVICTA, Polna Street 64, 81-740 Sopot, Poland; 3 InviMed Fertility Clinics, Rakowiecka Street 36, 02-532 Warsaw, Poland; faundez.ricardo@gmail.com; 4 Institute of Health Sciences, Collegium Salutis Humanae, University of Opole, Katowicka Street 68, 45-060 Opole, Poland; i.gilowska@gmail.com; 5 Clinical Center of Gynecology, Obstetrics and Neonatology in Opole, Reference Center for the Diagnosis and Treatment of Infertility, Reymonta Street 8, 45-066 Opole, Poland

**Keywords:** male infertility, seminal plasma, prostaglandins, antioxidant enzymes, oxidative stress biomarkers

## Abstract

Male infertility accounts for approximately 50% of reproductive failures, yet its diagnosis and understanding of underlying mechanisms remain limited. The present observational case–control study aimed to examine seminal plasma concentrations of prostaglandin E_2_ (PGE_2_), 6-keto-prostaglandin F_1α_ (6-keto-PGF_1α_), F_2_-isoprostane, superoxide dismutase 1 (SOD_1_), glutathione peroxidase 1 (GPX_1_), nitric oxide synthase 1 (NOS_1_), and their ratios, in fertile (n = 22, aged 24–45) and infertile (n = 250, aged 21–47) men, including analyses across specific infertile subgroups, using ELISA assays. Overall, no significant differences were observed between fertile and infertile men. However, subgroup analyses revealed notable differences: PGE_2_ levels were elevated in asthenozoospermic compared to normozoospermic infertile men (14.23 µg/mL vs. 3.52 µg/mL, *p* = 0.030), 6-keto-PGF_1α_ levels were higher in teratozoospermic compared to azoospermic individuals (184.97 ng/mL vs. 102.49 ng/mL, *p* = 0.040), and the PGE_2_/6-keto-PGF_1α_ ratio showed the greatest intergroup variability. Correlation analyses indicated associations between antioxidant enzymes, prostaglandins and standard semen parameters. These findings provide novel insights into seminal plasma biochemistry and highlight specific parameters and mechanisms that may contribute to functional impairments in infertile men.

## 1. Introduction

Infertility, defined as the inability to achieve natural conception after at least 12 months of regular unprotected intercourse, is becoming an increasingly prevalent health concern of the 21st century, affecting approximately 17.5% of couples worldwide according to the latest data [1,2]. The male factor plays a crucial role, contributing to nearly 50% of reproductive failures [3]. Nevertheless, the diagnosis, treatment, and prevention of male infertility, along with elucidation of its underlying mechanisms, remain insufficiently understood and demand further comprehensive investigation.

Standard semen analysis remains one of the few routinely available diagnostic tools for evaluating male infertility. Its primary aim is to assess key sperm parameters, including concentration, motility, viability, and morphology [1,4]. However, despite its clinical relevance, semen analysis often fails to reveal the underlying etiology of reproductive dysfunction and, in cases of idiopathic infertility where semen parameters fall within reference ranges, is almost entirely uninformative [5].

In addition to the limited diagnostic methods, clinicians face the additional challenge of multiple contributing factors to male infertility, including endocrinological, immunological, lifestyle-related, and environmental factors [3,6]. Among them, particular attention should be given to oxidative stress (OS), which arises from an imbalance between the production of reactive oxygen species (ROS), such as hydrogen peroxide, superoxide anion, hydroxyl radical, etc., and the antioxidant defense mechanisms [6,7,8,9]. This imbalance disrupts endocrine regulation and testicular function, impairs spermatogenesis, and activates inflammatory pathways, collectively reducing sperm production, quality, and fertilizing capacity [7,10,11]. Although small amounts of ROS are required to initiate essential sperm functions such as capacitation and the acrosome reaction, excessive ROS levels can also directly impair sperm function by inducing sperm DNA fragmentation, protein oxidation and lipid peroxidation of the sperm membranes, which are highly susceptible to ROS-induced damage due to their high content of polyunsaturated fatty acids (PUFAs) [6,7,8,9,12,13,14,15,16]. As essential constituents of sperm membranes, PUFAs play a crucial role in maintaining membrane fluidity, flexibility, stability, and overall structural integrity, which are crucial for proper sperm function [12,17,18]. Lipid peroxidation not only compromises the integrity of the sperm plasma membrane, affecting sperm viability, motility, and morphology, but also leads to the release of PUFAs into the seminal plasma, where they might be further metabolized into PUFA-derived compounds, including arachidonic acid (AA) eicosanoids with pro- or anti-inflammatory activity, such as prostaglandins (i.e., prostaglandin E_2_ (PGE_2_), prostacyclin (PGI_2_)), leukotrienes, thromboxanes, etc., as well as non-enzymatically formed isoprostanes (IsoPs) (i.e., F_2_-isoprostane), which may serve as markers of oxidative damage [7,12,19,20]. Despite the well-established biological functions of eicosanoids, their role in male infertility diagnostics, treatment, and/or prevention, as well as the mechanisms by which they are associated with semen quality and reproductive health, remain poorly understood [12,21].

Seminal plasma plays a crucial role in the proper maturation, function, fertilization capacity, and protection of spermatozoa [22,23]. Increasing evidence identifies it as a valuable source of biomarkers and a key medium for elucidating the molecular and cellular mechanisms underlying male infertility [24,25,26]. As a highly complex environment, it contains a wide array of chemical compounds essential for sperm viability and for the proper progression of processes leading to fertilization, including lipids, ions, cell-free DNA, RNA, microRNA, peptides, proteins, oligosaccharides, fatty acids, hormones, cytokines, prostaglandins, enzymes, exosomes, and many others [24,25]. It also possesses a well-developed antioxidant system, which protects germ cells from ROS-induced damage [7,8,27]. Enzymatic antioxidants include different isoforms of superoxide dismutase (SOD), glutathione peroxidase (GPX), nitric oxide synthase (NOS), and others [8,28,29,30,31,32,33]. Together with non-enzymatic antioxidants (i.e., ascorbic acid and glutathione), these molecules constitute an integrated antioxidant system that safeguards redox homeostasis, whose contribution to male fertility is considered indispensable [7,8]. SOD_1_ (CuZnSOD) catalyzes the dismutation of superoxide radicals into hydrogen peroxide and molecular oxygen, serving as a first line of defense against ROS and constituting the predominant SOD isoform in seminal plasma, accounting for approximately 75% of total SOD activity in this fluid [34,35,36]. By reducing hydrogen peroxide to water, GPX_1_ limits ROS accumulation [37]. Therefore, it could potentially safeguard sperm membranes and contribute to the preservation of their structural integrity, motility, and overall viability, as supported by studies showing that seminal plasma GPX and SOD protect sperm phospholipids and phospholipid-bound fatty acids from oxidative damage in normozoospermic men [38]. In turn, NOS_1_ contributes to redox regulation by producing controlled levels of nitric oxide (NO), a signaling molecule involved in sperm capacitation, motility, and acrosome reaction; however, excessive NO production impairs mitochondrial function, induces DNA damage, and contributes to sperm dysfunction [32,39,40]. Although NOS_1_ is traditionally characterized as a neuronal isoform, some studies indicate its expression in testicular, accessory reproductive tissues, and spermatozoa [41,42,43].

The present observational case–control study aimed to evaluate differences in the concentrations of PGE_2_, 6-keto-prostaglandin F_1α_ (6-keto-PGF_1α_, stable metabolite of PGI_2_), F_2_-isoprostane, SOD_1_, GPX_1_, NOS_1_ as well as the ratios: NOS_1_/SOD_1_, NOS_1_/GPX_1_, GPX_1_/SOD_1_ and PGE_2_/6-keto-PGF_1α_ in seminal plasma from fertile and infertile men, including comparisons between subgroups of infertile individuals for the purpose of evaluating the utility of the investigated parameters as biomarkers of male infertility. To the best of our knowledge, the present study is the first to determine the concentrations of antioxidant enzymes and their ratios in the seminal plasma of fertile and infertile men. Moreover, the specific isoforms investigated herein have not been previously assessed in this context. Correlations between the examined parameters and standard semen characteristics were also assessed. Building on our previous study [44], in which we reported alterations in specific PUFA concentrations in seminal plasma between fertile and infertile men and demonstrated their associations with standard semen parameters and potential diagnostic and functional relevance, the present work extends these findings by investigating the relationships between the examined parameters and seminal plasma PUFA concentrations. The purpose of such analyses was to elucidate potential mechanisms underlying reduced male fertility by examining how the concentrations of selected parameters in seminal plasma are interrelated and how they may influence semen parameters and the composition of seminal plasma, ultimately affecting male reproductive potential.

## 2. Materials and Methods

### 2.1. Sample Collection

Seminal plasma samples were collected from infertile men (n = 250; age range: 24–45 years) and fertile men (n = 22; age range: 21–47 years) in collaboration with the Clinical Center of Gynecology, Obstetrics and Neonatology in Opole (Poland) and the InviMed Fertility Clinics located in Warsaw and Wroclaw (Poland).

For the infertile men, eligibility required an inability to achieve conception with the same female partner for a minimum of 24 months. For both infertile and fertile participants, exclusion criteria comprised any medical history of conditions such as malignancies, mumps orchitis, adenomas, cardiovascular disease, nephritis, hepatitis, diabetes mellitus, eating disorders, genitourinary inflammation, Klinefelter’s syndrome, cryptorchidism, testicular torsion, varicocele, sexually transmitted infections, obesity, neurological disorders, or tuberculosis. Individuals with a history of surgical interventions or other injuries involving the genitourinary tract, scrotum, or groin region were likewise excluded. At the time of enrollment, all participants were required to have no current acute infectious diseases with high fever, no leukocytospermia, and/or the presence of bacteria in semen. For the fertile reference group, the additional criterion was the confirmed fatherhood of at least one child younger than three years. Data on potential confounding factors, such as dietary patterns, body mass index (BMI), and smoking habits, were not obtained during recruitment. This omission stemmed from the retrospective and exploratory design of the study and, consequently, such parameters were neither applied as eligibility criteria nor statistically controlled in subsequent analyses.

The ejaculates were obtained by masturbation into sterile containers following a period of 3–5 days of sexual abstinence. After liquefaction (≤60 min at 37 °C), a routine semen analysis was performed, including the evaluation of semen volume, pH, and sperm viability. Computer-assisted semen analysis (CASA) was carried out using the SCA Motility and Concentration software (version 6.5.0.5, Microptic SL, Barcelona, Spain) to determine additional parameters, namely total sperm count, sperm concentration, total motility, progressive motility, and sperm morphology. The concentration of morphologically abnormal spermatozoa was subsequently calculated based on sperm concentration and the percentage of morphologically normal spermatozoa. Following standard semen assessment, samples were centrifuged at 3500× *g* for 10 min at room temperature to obtain seminal plasma, which was then divided into smaller portions and stored at –86 °C in the Wroclaw Medical University Biobank until analyses. All specimens were processed and evaluated under anonymized conditions.

The study population was stratified into subgroups based on conventional semen analysis following the 2021 WHO guidelines [1]. Detailed definitions and sample sizes for each subgroup are summarized in Table 1. The characteristics of semen parameters in groups of infertile patients have been previously reported [44].

Written informed consent was obtained from all participants before enrollment in the study. The study protocol received approval from the Bioethics Committee for Human Research at Wroclaw Medical University (KB-739/2022, KB-201/2024, and KB-580/2024). All procedures for sample collection and handling were conducted in accordance with the International Council for Harmonisation Good Clinical Practice (ICH-GCP) guidelines and adhered to the ethical principles of the II^nd^ Declaration of Helsinki.

### 2.2. Assay Measurements

The concentrations of all analyzed parameters were measured in duplicate using ELISA assays performed according to the manufacturers’ protocols (Table 2). The measurements were conducted on a Mindray-96A reader (Mindray, Shenzhen, China).

### 2.3. PUFAs Extraction and Analysis

The determination of PUFA concentrations (linoleic acid (LA), α-linolenic acid (ALA), γ-linolenic acid (GLA), arachidonic acid (AA), eicosapentaenoic acid (EPA), and docosahexaenoic acid (DHA)) in seminal plasma was performed in our previous study, using gas chromatography-tandem mass spectrometry (GC-MS/MS) as described therein [44]. In brief, PUFAs were extracted from 75 µL of seminal plasma using a modified n-hexane/ethanol mixture (5:2, *v*/*v*) and subsequently converted to fatty acid methyl esters (FAMEs) using methanol and acetyl chloride (1.5 mL and 200 µL, respectively) (85 °C for 3 h). Chromatographic separation and quantification were conducted using GC-MS/MS. Identification and quantification of individual PUFAs were achieved using external standards, and results were normalized to concentrations per milliliter of seminal plasma (µg/mL) [44].

### 2.4. Statistical Analysis

Statistical analyses were conducted using Statistica 13.3 PL (StatSoft Poland Sp. z o.o., Kraków, Poland) and GraphPad Prism (GraphPad Software, version 10.4.1, San Diego, CA, USA). Results are presented as medians (Me) with interquartile ranges (Q1–Q3). The distribution of data was evaluated with the Shapiro–Wilk test. As most variables deviated from a Gaussian distribution, nonparametric statistical tests were applied. Comparisons of seminal plasma concentrations of individual parameters, as well as their calculated ratios, between fertile and infertile men were performed using the Mann–Whitney U test. To examine differences among subgroups within the infertile cohort, the Kruskal–Wallis ANOVA was employed, followed by Dunn’s post hoc test. Correlations between the investigated parameters, conventional semen characteristics, and PUFA concentrations were assessed using Spearman’s rank test. The strength of correlations was classified according to standard interpretative thresholds: negligible (|R| ≤ 0.2), weak (0.2 < |R| ≤ 0.4), moderate (0.4 < |R| ≤ 0.7), strong (0.7 < |R| ≤ 0.9), and very strong (|R| > 0.9). A two-tailed *p*-value of <0.05 was considered significant.

The outcomes of analyses are presented in Tables and Figures in the main text and in the Supplementary Materials (Tables S1–S6, Figures S1 and S2).

## 3. Results

### 3.1. Comparison of the Concentrations of Examined Parameters in Seminal Plasma Between Infertile Patients and Fertile Men

Comparative analysis of examined parameters between fertile men and infertile men revealed no significant differences. Median concentrations of PGE_2_, 6-keto-PGF_1α_, F_2_-isoprostane, SOD_1_, GPX_1_, NOS_1,_ and their ratios were comparable between groups. The obtained results are presented in Table 3.

### 3.2. Comparison of the Concentrations of Examined Parameters in Seminal Plasma Between Subgroups of Men

**Figure 1:**
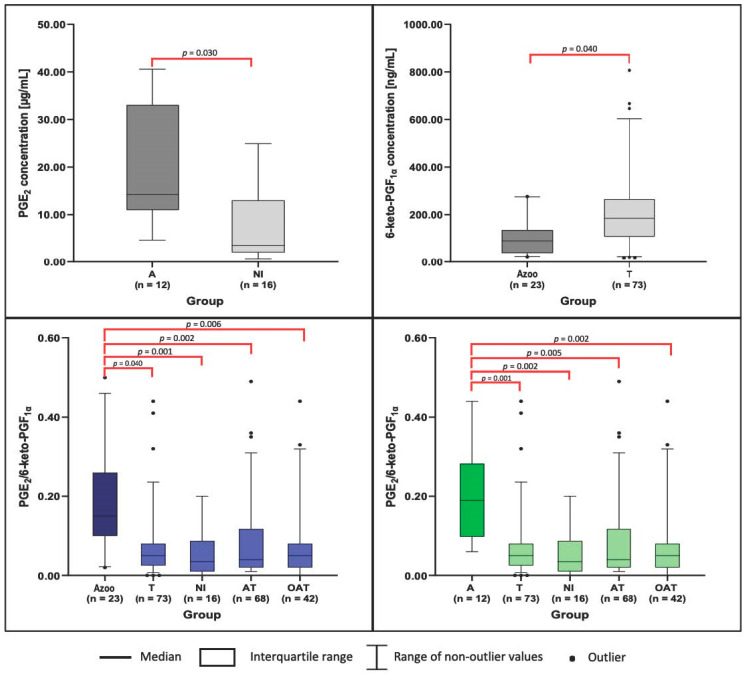
Comparison of PGE_2_, 6-keto-PGF_1α_ concentrations and their ratio between groups of infertile men using box plots. A two-tailed *p*-value of <0.05 was considered significant—*p*-values from Dunn’s test are provided. 6-keto-PGF_1α_—6-keto-Prostaglandin F_1α_, A—Asthenozoospermic group, AT—Asthenoteratozoospermic group, Azoo—Azoospermic group, NI—Normozoospermic infertile group, OAT—Oligoasthenoteratozoospermic group, PGE_2_—Prostaglandin E_2_, T—Teratozoospermic group, n—number of participants.

Analysis of seminal plasma parameters across subgroups of men revealed several differences. PGE_2_ concentrations were significantly elevated in the A group compared to NI. In addition, 6-keto-PGF_1α_ levels were higher in the T group relative to Azoo. The PGE_2_/6-keto-PGF_1α_ ratio demonstrated the most pronounced intergroup variability, with significant differences observed across multiple subgroup comparisons (T vs. A, T vs. Azoo, A vs. NI, A vs. AT, A vs. OAT, Azoo vs. NI, Azoo vs. AT, and Azoo vs. OAT). These findings are summarized in Table 4 and illustrated in Figure 1. No other parameters showed significant variation between the compared subgroups.

### 3.3. Correlation Analysis

**Figure 2:**
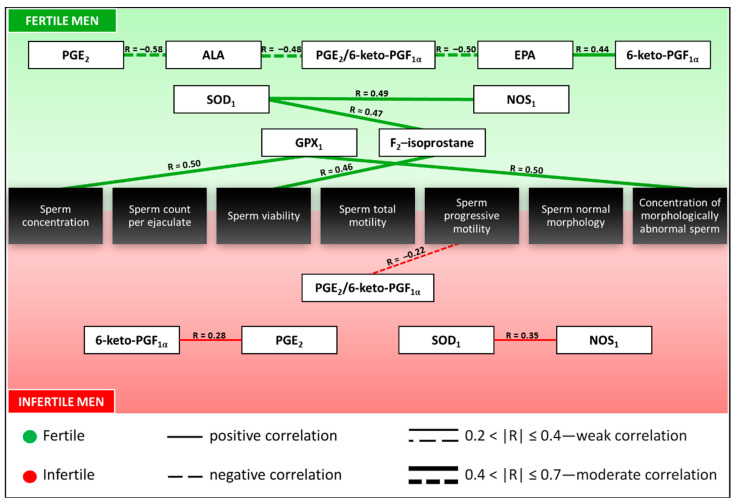
A graphical representation of the correlations between the examined parameters. 6-keto-PGF_1α_—6-keto-Prostaglandin F_1α_, ALA—α-linolenic acid, EPA—eicosapentaenoic acid, GPX_1_—Glutathione Peroxidase 1, NOS_1_—Nitric Oxide Synthase 1, PGE_2_—Prostaglandin E_2_, SOD_1_—Superoxide Dismutase 1, R—Spearman’s rank coefficient. PUFA concentrations (ALA, EPA) were determined in our previous study [44].

The significant correlations observed between examined parameters, standard semen analysis parameters, and PUFA concentrations in fertile and infertile men are shown in Table 5 and Figure 2.

In the fertile group, moderate positive correlations were detected between GPX_1_ concentrations and both sperm concentration and the concentration of morphologically abnormal sperm. Additionally, F_2_-isoprostane concentrations correlated moderately and positively with sperm viability. Conversely, PGE_2_ concentrations exhibited a moderate negative correlation with ALA levels, whereas the PGE_2_/6-keto-PGF_1α_ ratio showed moderate negative correlations with both ALA and EPA concentrations. 6-keto-PGF_1α_ levels showed a moderate positive correlation with EPA concentrations. Additional moderate positive associations were observed between F_2_-isoprostane and SOD_1_, as well as between NOS_1_ and SOD_1_ concentrations.

In the infertile group, weak negative correlations were noted between the PGE_2_/6-keto-PGF_1α_ ratio and sperm progressive motility, while a weak positive correlation was present between PGE_2_ and 6-keto-PGF_1α_ concentrations. A moderate positive correlation was also detected between NOS_1_ and SOD_1_ levels.

Comprehensive analyses, including non-significant results, are provided in the Supplementary Materials (Tables S1–S6).

## 4. Discussion

Male infertility is often, though not always, associated with abnormal semen parameters, such as sperm concentration, motility, viability, and/or morphology [5,45]. Impaired semen quality and reduced male reproductive potential may arise from various factors, including a disrupted oxidative-antioxidative balance in semen [7,10,15,27,46]. ROS in semen may originate from spermatozoa, especially those immotile/morphologically abnormal, and leukocytes, and although their generation is a normal physiological process, excessive production is detrimental and has been associated with male infertility, showing positive correlations with abnormal sperm concentration, motility, and morphology [7,10]. Moreover, OS may be a result of exogenous factors such as infections, smoking, or environmental toxins, etc. [6,12,47]. Previous studies have shown that elevated levels of ROS are detectable in the semen of approximately 40% of infertile men [48,49].

Excessive ROS generation promotes lipid peroxidation within sperm membranes and, through the activation of phospholipase A_2_, facilitates the release of lipid components into the seminal plasma [6,7,8,27,46]. These include PUFAs, particularly omega-6 fatty acids, among which AA is one of the predominant representatives [7,8]. AA is enzymatically metabolized via cyclooxygenase (COX-1 and COX-2), lipoxygenase (LOX), and cytochrome P450 (CYP 450) enzymes, leading to the generation of eicosanoids such as prostaglandins, leukotrienes, and thromboxanes [12,50,51]. Although the general biological functions of eicosanoids are relatively well established, their specific roles in male fertility/infertility, as well as the mechanisms through which they may affect semen quality and reproductive potential, remain insufficiently characterized. However, as indicated by previous studies, prostaglandin concentrations in seminal plasma are relatively high and may vary depending on fertility disorders and be associated with sperm parameters [52,53,54,55,56]. Isidori et al. [57] observed that prostaglandin levels in semen from infertile men may be either elevated or decreased, suggesting that both excessive and insufficient concentrations could be detrimental. Other studies indicated that maintaining appropriate levels of certain prostaglandins (i.e., PGE_2_) is essential, as they play a crucial role in fertilization processes, including the establishment of maternal tolerance, optimal embryo implantation, and placental development [12,58].

Prostaglandins E are present at the highest concentrations in seminal plasma, and their predominant representative is PGE_2_ [52,59,60,61]. Several reports available in the literature suggested a potential diagnostic value of prostaglandin E determination in the context of male infertility, as its lower concentration was observed in the seminal plasma of infertile men compared to fertile controls [53,54,62]. However, most of these studies date back approximately 50 years, which, considering evolving diagnostic criteria and analytical methodologies, limits their direct applicability to contemporary conditions. In these investigations, the reported mean seminal plasma concentrations of prostaglandin E ranged from 22.1 µg/mL to 73.2 µg/mL [52,53,54]. Nevertheless, it should be noted that the PGE_2_, examined in the present study, is only a part of the prostaglandin E group, which comprises several isomers, including also PGE_1_ and PGE_3_ [12]. Recently, Amor et al. [63], using the same analytical methodology as in the present study, measured seminal plasma PGE_2_ levels in men attending a fertility clinic and reported substantially lower concentrations (1854 ± 63.7 pg/mL) than those obtained in any of our analyzed groups. The most likely explanation for this discrepancy lies in differences in sample storage conditions. Our samples were stored at −86 °C, whereas Amor et al. [63] stored theirs at −20 °C. As reported by Cao et al. [64], storage conditions exert a critical influence on the stability of prostaglandins, with temperatures above −80 °C potentially leading to partial degradation of these eicosanoids. Another prostaglandin of interest in the context of male infertility might be PGI_2_, which undergoes rapid metabolism to the stable metabolite 6-keto-PGF_1α_ [50,61,65].

Regarding differences in seminal plasma PGE_2_, Huleihel et al. [66] demonstrated higher concentrations in fertile donors than in the infertile OAT group (19.67 µg/mL vs. 7.67 µg/mL), while Schlegel and Meyer [65] reported reduced levels of both PGE_2_ (24.0 ± 6.6 µg/mg protein) and PGI_2_ (5.6 ± 1.4 pg/mg protein) in men with decreased sperm motility compared with those showing motility above 40%. In contrast, more recent data from 2023 by Chen et al. [67] indicated comparable seminal plasma concentrations of PGE_2_ (8.00 ± 1.17 µg/L vs. 8.26 ± 0.94 µg/L) and PGI_2_ (2.48 ± 2.07 µg/L vs. 1.78 ± 2.30 µg/L) between fertile and infertile men, with no significant intergroup differences. Moreover, to the best of our knowledge, only Lewy et al. [62] have examined differences in seminal plasma 6-keto-PGF_1α_ concentrations between fertile (198 ± 54 pmol/mL) and infertile (128 ± 54 pmol/mL) men, and found no significant differences. Despite methodological discrepancies, a comparable lack of significant differences in seminal plasma concentrations of PGE_2_ or 6-keto-PGF_1α_ was observed in the present study when comparing fertile and infertile groups without subdivision into specific abnormalities of sperm parameters. However, although we did not observe the same differences as reported by Huleihel et al. [66] and Schlegel and Meyer [65], the analysis of examined parameters across the defined subgroups of infertile men revealed significant differences. PGE_2_ concentrations were markedly elevated in the A group compared with the NI subgroup. Previous studies have suggested that inflammatory factors and OS contribute to the etiology of asthenozoospermia, and both have been associated with increased COX-2 expression [68,69]. Furthermore, Salvolini et al. [70] demonstrated that the levels of COX-2 are higher in spermatozoa from the asthenozoospermic group compared to the normozoospermic group. Taken together, these findings suggest that the elevated levels of PGE_2_ observed in asthenozoospermia may be attributable to enhanced COX-2 expression. Moreover, ROS production increases with the proportion of immotile spermatozoa, and OS may facilitate the liberation of PUFAs from the sperm membrane, thereby augmenting the availability of precursors such as AA for prostaglandin biosynthesis, including PGE_2_ [7,12]. Additionally, in the present study, seminal plasma 6-keto-PGF_1α_ levels were lowest in the Azoo group compared with all other groups, although the difference reached statistical significance only in comparison with the T group. The most likely explanation for this observation might be the absence of spermatozoa in the Azoo group, which limits the availability of AA from sperm membranes. In the teratozoospermic group, greater damage to sperm membranes may increase AA availability for enzymatic conversion, leading to higher production of PGI_2_ and consequently higher levels of 6-keto-PGF_1α_. Given the absence of a similar observation for PGE_2_, it is plausible that the sources of prostaglandins in seminal plasma differ and contribute to their presence to varying degrees. It can be hypothesized that while PGI_2_ may derive predominantly from spermatozoa, PGE_2_ has additional sources, such as seminal vesicles, which could potentially explain the substantially higher concentrations of PGE_2_ compared with PGI_2_ or 6-keto-PGF_1α_ in seminal plasma [71]. The above hypotheses may also explain the observed differences in the PGE_2_/6-keto-PGF_1α_ ratio between groups. In the Azoo group, the complete absence of spermatozoa likely results in reduced PGI_2_ production, whereas PGE_2_ continues to be produced by the seminal vesicles, leading to a higher PGE_2_/6-keto-PGF_1α_ ratio compared with the T, NI, AT, and OAT groups. Interestingly, no significant differences were observed between the Azoo and A groups. Although asthenozoospermia is associated with increased PGE_2_ production [65], the absence of morphological sperm damage in A group may limit AA availability for 6-keto-PGF_1α_ synthesis. Consequently, the PGE_2_/6-keto-PGF_1α_ ratio remains comparably high to that observed in the azoospermic group, where the absence of spermatozoa eliminates the potential primary source of 6-keto-PGF_1α_. Increased COX-2 expression associated with asthenozoospermia may potentially explain why higher PGE_2_/6-keto-PGF_1α_ ratios were observed in the A group compared with T, NI, AT, and OAT. Remarkably, the mixed groups differed significantly from the A group. This may be due to morphological abnormalities of spermatozoa that likely enhance 6-keto-PGF_1α_ production, which results in a lower PGE_2_/6-keto-PGF_1α_ ratio compared with the A group. Similarly, the observed differences between the A and NI groups may stem from the fact that the NI group examined in our study exhibited a high proportion of damaged spermatozoa (44–96%), which further supports the interpretation of the differences observed between group A and groups in which the percentage of sperm with malformations was higher than 4%. It is also worth noting that differences between the A/Azoo groups and the F and OT groups were likewise observed, although insignificant. The present study establishes an interesting research direction, as confirmation of the hypotheses mentioned above could not only identify the sources of prostaglandins in seminal plasma but also evaluate the potential value of the seminal plasma PGE_2_/6-keto-PGF_1α_ ratio as a parameter reflecting not only COX-2 enzymatic activity and sperm functionality, but also inflammatory processes and oxidative stress in seminal plasma, which may hold diagnostic and therapeutic significance.

Beyond its enzymatic metabolism, AA can be non-enzymatically oxidized, resulting in the formation of IsoPs, especially F_2_-isoprostane [72]. IsoPs not only exhibit pro-inflammatory properties and act as potential biomarkers of oxidative stress but also function as modulators of vascular activity, influencing vasoconstriction, vasodilation, and platelet aggregation [73,74]. Currently, F_2_-isoprostane is widely recognized as the most reliable marker of lipid peroxidation and is more commonly used to assess oxidative status across a range of human diseases [75,76]. With respect to male infertility, recent studies have demonstrated that F_2_-isoprostane can be identified both within sperm membranes and in seminal plasma, where it may serve as a diagnostic biomarker and correlate with semen quality as well as overall reproductive potential [77,78,79]. Nevertheless, its exact pathophysiological role and clinical significance remain incompletely understood, underscoring the need for further in-depth research.

In the present study, no significant differences in seminal plasma F_2_-isoprostane levels were observed between men with proven fertility and infertile men, both in the analysis of the entire infertile cohort and when subdivided into subgroups. These findings are partially consistent with those reported by Collodel et al. [77] and Longini et al. [80], who also found no differences between the fertile and NI group, but reported significantly higher F_2_-isoprostane levels in infertile patients with varicocele. Similarly, in another study, Collodel et al. [78] additionally confirmed no differences in F_2_-isoprostane levels between the fertile and NI group, whereas concentrations of this parameter were elevated in groups with varicocele and leukocytospermia. Moretti et al. [79,81] observed higher F_2_-isoprostane levels in men with varicocele and genitourinary infections compared with fertile men; however, in the NI cohort, results were inconsistent—one study reported no significant differences [79], whereas another demonstrated higher levels compared with fertile men [81]. Collectively, these observations suggest that elevated F_2_-isoprostane concentrations are more likely associated with specific pathologies, such as varicocele or genitourinary infections, rather than abnormal sperm parameters and idiopathic infertility itself.

As previously mentioned, to counteract OS in semen and thereby prevent sperm damage and the generation of metabolites harmful to male fertility, seminal plasma contains a highly specialized antioxidant system, comprising different isoforms of enzymes such as SOD, GPX, and NOS [8,30,32,33]. Despite extensive research on seminal plasma antioxidants, including SOD, GPX, and NOS, in the context of male infertility, most studies have assessed only their enzymatic activity, whereas in numerous pathological conditions, their concentrations were proven to be relevant for both diagnostic and mechanistic insights [82,83,84,85,86,87]. Moreover, freezing and thawing samples (as was done in the mentioned studies) can affect enzyme activity, and these effects depend on many factors, including the type of sample, the type of enzyme, the freezing rate, and the number of freeze–thaw cycles, which suggests that enzyme activity may not be the most reliable parameter in studies of this kind [88,89]. To date, only a single study has reported on SOD concentration in seminal plasma [90]. Moreover, few studies have examined the presence and concentrations of specific isoforms, particularly for GPX and NOS, leaving their isoform-specific roles in seminal plasma almost totally unexplored. Therefore, the discussion refers succinctly to their enzymatic activity to underscore the observed differences.

It was generally believed that seminal plasma from infertile men exhibits lower antioxidant activity compared with that of fertile men, without stratification into specific subgroups [7]. Nevertheless, recent findings have frequently demonstrated considerable inconsistencies. Some studies reported lower seminal plasma SOD activity in infertile compared with fertile men [32,91,92,93,94], while others found no significant differences [40,95]. In contrast, a few authors observed higher SOD activity in the seminal plasma of infertile men, which was interpreted as a compensatory response to oxidative stress [96,97]. Several studies consistently demonstrated lower seminal plasma GPX activity [7,40,94,98] and increased NOS activity [32,99] in infertile men compared with fertile controls. In the context of comparisons between fertile and infertile men, the present study represents the only report investigating the concentrations of the enzymes SOD, GPX, and NOS in seminal plasma, specifically focusing on their individual isoforms (SOD_1_, GPX_1_, and NOS_1_). No significant differences were observed between the analyzed groups, which, when considered alongside previous findings on the activity of these enzymes, may suggest that male infertility is not directly associated with alterations in the concentrations of specific enzyme isoforms, but rather with their enzymatic activity. It is important to emphasize, however, that this study focused exclusively on selected enzyme isoforms, which considerably limits the ability to compare the obtained results with the existing literature. On the other hand, the results of the present study contribute to expanding the scope of information on the associations between the concentration of parameters selected for the present research and male infertility. The division of men into specific subgroups based on semen parameter abnormalities also did not reveal differences. Notably, only Yin et al. [90] examined seminal plasma concentrations of SOD and reported no significant differences in its levels between normozoospermic and OAT groups (44.15 pg/mL (31.51–57.00) vs. 43.89 pg/mL (34.78–57.79), respectively). In the present study, no differences were observed in SOD_1_ levels. However, a direct comparison with the results obtained by Yin et al. [90] is not possible, as those authors assessed total SOD concentrations, not a specific SOD_1_ isoform. An important observation is that the total SOD concentrations reported by Yin et al. [90] were markedly lower compared to our results obtained for a single isoform of this enzyme. This substantial discrepancy may stem from differences in sample preparation procedures. Specifically, Yin et al. [90] froze the entire semen sample and subsequently obtained seminal plasma after thawing, while in our study, seminal plasma was separated from spermatozoa before freezing. Several authors reported no differences in total seminal plasma SOD activity between normozoospermic and other groups of men, including A, T, AT, oligoasthenozoospermic (OA), and OAT [100,101,102,103], whereas others observed either lower [36,95,103,104] or higher [102,105] SOD activity in specific subgroups compared with the normozoospermic group. Only Chyra-Jach et al. [103] specifically assessed seminal plasma SOD_1_ activity and found no significant differences between normozoospermic and oligozoospermic (O), A, and OA groups of men. When proven fertile men were used as controls, SOD activity in seminal plasma was generally lower in infertile groups. Zelen et al. [106] reported reduced SOD activity in NI, O, A, and T groups compared with fertile controls. Ramya et al. [107] similarly found lower SOD levels in O, OA, and T men, but no differences in NI or A subjects in comparison to fertile men. In contrast, Ammar et al. [108] observed comparable SOD activity between the T group and fertile men. Findings on GPX activity are also inconsistent. While some studies reported decreased [36,104,108], others found no difference [31] or increased GPX activity [103,105] in seminal plasma from certain infertile subgroups compared to normozoospermic or fertile men. Limited data suggest that NOS activity is elevated in A group of men, reduced in OA and T patients, and unchanged in NI men compared with proven fertile controls [107]. Given the lack of conclusive evidence in previous studies, the present study aimed to assess whether the seminal plasma concentrations of individual enzymes could serve as more informative parameters. However, in this context, our analysis did not reveal any significant differences in levels of these parameters between the examined groups. Nevertheless, given the observational design of the study and the limited sample size in the fertile group, additional analyses with larger cohorts, especially fertile male individuals, are warranted to validate the findings presented herein.

An important finding from our analyses is that the concentrations of the selected parameters show no significant differences between fertile and infertile men. This may reflect a true absence of variation in the measured parameters, suggesting that, at least for these markers, fertility status alone does not exert a major influence. However, alternative explanations cannot be excluded. The relatively small sample size of the fertile group may have limited the statistical power to detect subtle differences, and potential confounding factors, such as BMI and lifestyle variables (e.g., diet, smoking status), were not accounted for in the present study.

Previous studies reported inconsistent associations between seminal plasma prostaglandins, OS markers, antioxidant enzymes, and standard semen analysis parameters. Bendvold et al. [55] reported a negative correlation between seminal plasma PGE concentrations and sperm count but not motility, whereas Isidori et al. [57] observed that both elevated and reduced seminal plasma PGE levels were associated with decreased sperm motility and concentration. In contrast, Al-Maliki et al. [109] found no significant associations between seminal plasma PGE_2_ concentrations and semen parameters in infertile men. Chen et al. [67] demonstrated that, in normozoospermic men, seminal plasma PGE_2_ levels correlated positively with sperm concentration but not with motility. With respect to PGI_2_, both Schlegel et al. [65] and Chen et al. [67] reported no correlation between seminal plasma PGI_2_ concentrations and either sperm motility or concentration. In the present study, no correlations were observed between seminal plasma PGE_2_ or the stable metabolite of PGI_2_, 6-keto-PGF_1α_, and semen parameters. However, the seminal plasma PGE_2_/6-keto-PGF_1α_ ratio showed a weak but significant negative correlation with progressive motility in infertile men (R = –0.22, *p* < 0.001), suggesting that altered prostaglandin balance within seminal plasma may contribute to impaired sperm motility. Regarding OS markers, previous studies reported conflicting findings for seminal plasma F_2_-isoprostane concentrations, ranging from no correlation with semen parameters [77,80] to weak or moderate negative associations with sperm motility, progressive motility, morphology, and viability but not concentration [79,81]. Interestingly, in seminal plasma from our fertile group, F_2_-isoprostane concentrations correlated positively with sperm viability (R = 0.46, *p* = 0.030), possibly indicating a physiological redox balance supporting sperm function. Data regarding antioxidant enzymes were likewise heterogeneous and limited to measurements of their activities in seminal plasma. Some studies found no significant correlations between seminal plasma SOD, GPX, or NOS activities and semen quality parameters [28,33,90,100,103]. In contrast, other reports demonstrated positive associations between SOD activity and both sperm concentration and motility [20,29,36,92,93]. Similarly, seminal plasma GPX activity has been shown to correlate positively with sperm concentration and motility [36,110], although findings regarding sperm morphology remain inconsistent—no association between SOD/GPX activity and sperm abnormal morphology was observed by Atig et al. [36], whereas Crisol et al. [110] reported a significant positive correlation between GPX activity and normal forms of sperm. In the present study, seminal plasma GPX_1_ concentration correlated positively with sperm concentration (R = 0.50, *p* = 0.023) and with the concentration of morphologically abnormal sperm (R = 0.50, *p* = 0.021) in fertile men. These associations may suggest a compensatory role of GPX_1_ in maintaining the overall sperm output and controlling reactive oxygen species within seminal plasma, thereby protecting spermatozoa from oxidative damage.

As previously described, ROS induce the release of PUFAs from sperm membranes into seminal plasma, where they can be transformed into prostaglandins and/or isoprostanes. This process may be counteracted by antioxidant enzymes present in seminal plasma. Therefore, exploring potential correlations between PUFAs, prostaglandins, F_2_-isoprostane, and antioxidant enzyme concentrations seems to be reasonable. Only Safarinejad [111] demonstrated that seminal plasma EPA and DHA concentrations were positively correlated with seminal plasma SOD-like activity. In the present study, other correlations were shown. In fertile men, moderate negative correlations were observed between PGE_2_ and ALA, as well as between the PGE_2_/6-keto-PGF_1α_ ratio and both ALA and EPA, while 6-keto-PGF_1α_ showed a moderate positive correlation with EPA. ALA and EPA are omega-3 PUFAs, whereas AA (the precursor of PGE_2_ and 6-keto-PGF_1α_) belongs to the omega-6 family. The omega-6/omega-3 ratio within sperm membrane phospholipids is considered a critical determinant of sperm function and fertility, with an elevated ratio frequently associated with impaired reproductive outcomes [12]. The observed correlations likely reflect a competitive metabolic relationship, in which higher levels of omega-3 fatty acids (ALA and EPA) reduce the availability of omega-6 substrates such as AA to enzymes in the same biochemical reaction pathway, thereby limiting the synthesis of PGE_2_ in the ejaculate. On the other hand, a positive correlation observed between 6-keto-PGF_1α_ and EPA may reflect an EPA-driven modulation of COX-2 expression and eicosanoid synthesis, although this interpretation remains hypothetical and requires further investigation [112,113]. Moderate positive correlations were also noted between F_2_-isoprostane and SOD_1_, as well as between NOS_1_ and SOD_1_. In response to OS, reflected by elevated levels of F_2_-isoprostane, the production of antioxidant enzymes may increase, which might explain the positive correlation with SOD_1_, representing the first line of defense against ROS. The observed correlation between NOS_1_ and SOD_1_ may further indicate a coordinated interaction between these two systems in the oxidative stress response. Such interplay may constitute an adaptive defense mechanism aimed at maintaining redox homeostasis and preserving sperm function. In infertile men, only weak positive correlations were observed, specifically between PGE_2_ and 6-keto-PGF_1α_, and between NOS_1_ and SOD_1_. The former may reflect increased availability of AA and its subsequent conversion to prostaglandins, while the latter is weaker than in fertile men, which may indicate a less efficient antioxidant response.

The present study demonstrates several noteworthy strengths that enhance its scientific and clinical relevance. Although the differences in examined parameters between fertile (PGE_2_: 8.27 µg/mL, 6-keto-PGF_1α_: 155.14 ng/mL, F_2_-isoprostane: 204.03 ng/L, SOD_1_: 16.40 ng/mL, GPX_1_: 28.12 ng/mL, NOS_1_: 4.28 ng/mL) and infertile (PGE_2_: 7.45 µg/mL, 6-keto-PGF_1α_: 159.30 ng/mL, F_2_-isoprostane: 207.62 ng/L, SOD_1_: 20.31 ng/mL, GPX_1_: 32.04 ng/mL, NOS_1_: 4.15 ng/mL) men were not significant, a key contribution of the present study lies in establishing seminal plasma concentrations for the investigated parameters, as such values are absent from the current literature.

Importantly, the inclusion of multiple subgroups of male infertility, including idiopathic cases, and the use of rigorously selected fertile controls with confirmed fertility rather than merely normozoospermic men, mark a significant methodological advancement over most previous research that often relied on outdated WHO criteria and unrepresentative control populations. The study’s innovative approach—assessing the concentrations rather than the activities of seminal plasma antioxidant enzymes, and for the first time, characterizing their isoforms in the context of male infertility—provides a reliable and informative dataset, less susceptible to preanalytical variability. Furthermore, the integrative analysis of correlations between examined parameters offers a more holistic view of the potential pathways underlying male infertility. Nevertheless, several limitations must be acknowledged. The exploratory and observational nature of the study precludes causal inference, and the notable imbalance between fertile and infertile cohorts, particularly the small size of the fertile control group, constrains statistical robustness and increases the likelihood of sampling bias. The absence of detailed information on BMI and lifestyle-related covariates such as diet, nutritional supplementation, and smoking status limits the interpretability of the findings, as these factors are known to modulate seminal plasma composition, oxidative-antioxidative balance, and inflammatory activity. Elevated BMI, for example, has been associated with increased oxidative stress in seminal plasma [114]. Obesity contributes to systemic low-grade inflammation and mitochondrial dysfunction, which together promote excessive ROS production and reduce antioxidant defenses, including SOD and GPX activity in testicular and epididymal tissues; these alterations have been consistently linked to impaired semen quality [115]. Smoking has also been repeatedly associated with elevated ROS and nitric oxide concentrations in semen, along with reductions in seminal total antioxidant capacity and key antioxidant enzymes such as SOD and GPX [116,117,118,119,120]. In contrast, adherence to antioxidant-rich dietary patterns—characterized by higher intake of vitamins C and E, carotenoids, and polyphenols—has been shown to strengthen seminal antioxidant capacity, attenuate ROS generation, and reduce lipid peroxidation processes [121,122]. Extending this evidence on nutritional modulation of oxidative balance, Strzeżek et al. [123] demonstrated that PUFA supplementation can upregulate seminal SOD activity, underscoring the role of targeted dietary components in regulating redox homeostasis. Additionally, while the study examined key representatives of the antioxidant and lipid peroxidation pathways, the spectrum of analyzed parameters remains relatively narrow, which may overlook additional mechanistic contributors to male infertility.

Future research should focus on expanding the cohort size, especially the fertile group. Investigations into other AA derivatives, additional isoforms of antioxidant enzymes, and the role of COX-1 and COX-2 in prostaglandin biosynthesis within seminal plasma could provide crucial mechanistic insights. It would also be essential to elucidate the origin of prostaglandins in seminal fluid and to determine whether antioxidant enzyme concentrations are associated with broader aspects of male reproductive health beyond semen parameters, such as specific infertility-related pathologies. Integrating environmental and lifestyle data into future analyses will help disentangle confounding influences and could pave the way for diet- or lifestyle-based preventive interventions.

## 5. Conclusions

The present observational study found no significant differences in the concentrations of PGE_2_, 6-keto-PGF_1α_, F_2_-isoprostane, SOD_1_, GPX_1_, NOS_1,_ as well as the ratios: NOS_1_/SOD_1_, NOS_1_/GPX_1_, GPX_1_/SOD_1,_ and PGE_2_/6-keto-PGF_1α_ in seminal plasma from fertile and infertile men, indicating that their discriminative potential is limited for distinguishing fertile from infertile individuals. Nevertheless, our results suggest that some of these parameters may retain meaningful differentiating value when comparing specific subgroups of infertile men.

Distinct alterations in prostaglandin profiles among infertile subgroups, particularly in the PGE_2_/6-keto-PGF_1α_ ratio, suggest that dysregulated eicosanoid metabolism and inflammatory signaling may contribute to specific infertility etiologies. The observed differences between subgroups, including elevated seminal plasma PGE_2_ in A compared to the NI group and elevated seminal plasma 6-keto-PGF_1α_ levels in T vs. the Azoo group, likely reflect divergent COX-2–related mechanisms and imply that prostaglandins in seminal plasma may originate from multiple cellular sources, including spermatozoa and accessory glands. Confirmation of these patterns could establish the seminal plasma PGE_2_/6-keto-PGF_1α_ ratio as a potential indicator of COX-2 activity, sperm functionality, and inflammatory or oxidative processes with possible diagnostic relevance.

In conjunction with findings reported in the literature, the lack of differences in seminal plasma F_2_-isoprostane concentrations between the studied groups indicates that this parameter may be associated with specific pathologies, such as varicocele or genitourinary infections.

The present study, for the first time, quantified specific antioxidant enzyme isoforms in seminal plasma. However, the lack of group-dependent differences in seminal plasma SOD_1_, GPX_1,_ and NOS_1_ concentrations, together with previously reported inconsistencies in enzymatic activity data, implies that male infertility may depend more on the functional activity than on the abundance of these antioxidant enzymes.

In fertile men, positive correlations were observed between parameters: F_2_-isoprostane with sperm viability, GPX_1_ with sperm concentration and with morphologically abnormal sperm, SOD_1_ with F_2_-isoprostane and NOS_1,_ and 6-keto-PGF_1α_ with EPA. Negative correlations were noted between prostaglandins and PUFAs: PGE_2_ vs. ALA, PGE_2_/6-keto-PGF_1α_ vs. ALA and EPA. In the infertile group, PGE_2_/6-keto-PGF_1α_ negatively correlated with progressive motility, PGE_2_ positively with 6-keto-PGF_1α_, and NOS_1_ positively with SOD_1_. These findings highlight that oxidative stress, prostaglandin metabolism, and antioxidant defenses are interrelated, and their mutual dependencies are worth attention in the context of searching for the causes of infertility/reduced fertility or idiopathic infertility.

## Figures and Tables

**Table 1:** Classification of study groups based on semen parameters and fertility status.

Group	Abbreviation	N	Defining Criteria
Teratozoospermic	T	73	<4% sperm normal morphology
Asthenozoospermic	A	12	<32% sperm progressive motility
Azoospermic	Azoo	23	Complete absence of spermatozoa in the ejaculate
Infertile normozoospermic	NI	16	All semen parameters are within WHO reference limits
Asthenoteratozoospermic	AT	68	<32% sperm progressive motility and <4% sperm normal morphology
Oligoteratozoospermic	OT	16	Sperm concentration < 15 × 10^6^/mL and <4% sperm normal morphology
Oligoasthenoteratozoospermic	OAT	42	Sperm concentration < 15 × 10^6^/mL and <32% sperm progressive motility and <4% sperm normal morphology
Fertile	F	22	Proven fertility (≥1 child < 3 years of age)

**Table 2:** Characteristics of the assay kits used for the determination of the analyzed parameters.

Parameter	Manufacturer	Catalog No.	Assay Range	Lower Limit of Detection	Intra-Assay Precision	Inter-Assay Precision
PGE_2_	Cayman Chemical (Ann Arbor, MI, USA)	500141	15.60–2000.00 pg/mL	11.00 pg/mL	CV = 10.30%	CV = 3.60%
6-keto-PGF_1α_	Biorbyt (Cambridge, UK)	orb568161	0.02–1.00 ng/mL	0.01 ng/mL	CV = 6.20%	CV = 6.24%
F_2_-isoprostane	Sunlong Biotech (Hangzhou, China)	SL3468Hu	10.00–420.00 ng/L	1.00 ng/L	CV < 10%	CV < 12%
SOD_1_	BT LAB (Shanghai, China)	E4502Hu	0.50–180.00 ng/mL	0.24 ng/mL	CV < 8%	CV < 10%
GPX_1_	ELK Biotechnology (Sugar Land, TX, USA)	ELK1928	3.13–200.00 ng/mL	1.20 ng/mL	CV < 8%	CV < 10%
NOS_1_	BT LAB (Shanghai, China)	E0924Hu	0.10–40.00 ng/mL	0.05 ng/ml	CV < 8%	CV < 10%

CV—coefficient of variation, 6-keto-PGF_1α_—6-keto-Prostaglandin F_1α_, GPX_1_—Glutathione Peroxidase 1, NOS_1_—Nitric Oxide Synthase 1, PGE_2_ –Prostaglandin E_2_, SOD_1_—Superoxide Dismutase 1.

**Table 3:** Comparison of the concentrations of the investigated parameters and their ratios in seminal plasma between fertile and infertile men.

Parameter	Group		*p*-Value
	Fertile (n = 22)	Infertile (n = 250)	
PGE_2_ [μg/mL]	8.27 (3.18–32.17)	7.45 (3.56–15.48)	0.511
6-keto-PGF_1α_ [ng/mL]	155.14 (122.10–188.90)	159.30 (86.18–243.12)	0.916
F_2_-isoprostane [ng/L]	204.03 (193.35–221.70)	207.62 (189.23–237.79)	0.545
SOD_1_ [ng/mL]	16.40 (13.42–23.30)	20.31 (15.30–27.70)	0.095
GPX_1_ [ng/mL]	28.12 (17.65–37.47)	32.04 (21.76–50.91)	0.120
NOS_1_ [ng/mL]	4.28 (3.12–7.43)	4.15 (3.22–8.11)	0.775
NOS_1_/SOD_1_	0.24 (0.19–0.31)	0.20 (0.15–0.29)	0.115
NOS_1_/GPX_1_	0.18 (0.10–0.37)	0.15 (0.09–0.25)	0.501
GPX_1_/SOD_1_	1.45 (1.01–2.01)	1.57 (1.01–2.61)	0.487
PGE_2_/6-keto-PGF_1α_	0.08 (0.02–0.16)	0.06 (0.03–0.12)	0.556

The values are presented as medians and interquartile ranges (Q1–Q3). The Mann–Whitney U test was used to assess the differences between groups. A two-tailed *p*-value of <0.05 was considered significant. 6-keto-PGF_1α_—6-keto-Prostaglandin F_1α_, GPX_1_—Glutathione Peroxidase 1, NOS_1_—Nitric Oxide Synthase 1, PGE_2_—Prostaglandin E_2_, SOD_1_—Superoxide Dismutase 1, n—number of participants.

**Table 4:** Comparison of the concentrations of the investigated parameters and their ratios in seminal plasma between the examined groups of men.

	Group	T (n = 73)	A (n = 12)	Azoo (n = 23)	NI (n = 16)	AT (n = 68)	OT (n = 16)	OAT (n = 42)	F (n = 22)
Parameter									
PGE_2_ [μg/mL]		6.70 (4.01–13.02)	**14.23 ^a^** **(11.25–29.98)**	11.31 (4.72–38.85)	3.52 (2.11–12.48)	7.12 (3.09–16.73)	11.77 (4.17–23.35)	5.26 (3.05–11.76)	8.27 (3.18–32.17)
6-keto-PGF_1α_ [ng/mL]		**184.97 ^b^** **(108.18–265.36)**	102.49 (60.17–169.13)	89.78 (38.33–135.18)	209.06 (124.19–246.86)	162.94 (83.92–291.91)	192.87 (100.49–274.90)	147.24 (86.78–254.61)	155.14 (122.10–188.90)
F_2_-isoprostane [ng/L]		208.52 (181.32–247.67)	217.13 (204.54–222.06)	201.72 (193.48–227.31)	188.62 (164.38–215.34)	208.27 (192.67–238.67)	225.24 (199.54–255.73)	202.10 (185.53–237.79)	204.03 (193.35–221.70)
SOD_1_ [ng/mL]		19.25 (14.06–23.18)	20.05 (14.02–33.50)	21.62 (17.08–33.14)	24.34 (12.41–39.00)	22.09 (18.02–35.41)	15.38 (12.64–21.01)	21.22 (16.88–27.70)	16.40 (13.42–23.30)
GPX_1_ [ng/mL]		33.90 (23.29–51.86)	27.78 (20.13–47.37)	27.68 (19.39–74.65)	18.89 (15.40–41.11)	35.93 (26.68–62.19)	23.88 (16.07–34.14)	34.16 (22.53–44.67)	28.12 (17.65–37.47)
NOS_1_ [ng/mL]		4.29 (3.08–7.98)	3.54 (3.16–4.58)	3.66 (3.13–4.51)	6.47 (3.81–13.52)	4.16 (3.26–8.61)	4.94 (3.30–9.76)	4.20 (2.94–7.18)	4.28 (3.12–7.43)
NOS_1_/SOD_1_		0.22 (0.16–0.30)	0.18 (0.14–0.30)	0.16 (0.11–0.25)	0.18 (0.15–1.06)	0.20 (0.16–0.28)	0.23 (0.19–0.85)	0.18 (0.16–0.25)	0.24 (0.19–0.31)
NOS_1_/GPX_1_		0.15 (0.07–0.23)	0.16 (0.09–0.21)	0.15 (0.06–0.20)	0.27 (0.17–0.48)	0.15 (0.08–0.28)	0.19 (0.13–0.51)	0.14 (0.08–0.22)	0.18 (0.10–0.37)
GPX_1_/SOD_1_		1.70 (1.12–3.26)	1.43 (0.89–2.26)	1.62 (0.90–2.41)	1.07 (0.42–1.75)	1.63 (1.01–3.19)	1.34 (1.08–1.98)	1.55 (0.87–2.21)	1.45 (1.01–2.01)
PGE_2_/6-keto-PGF_1α_		**0.05 ^c,d^** **(0.03–0.08)**	**0.19 ^e,f,g^** **(0.13–0.27)**	**0.15 ^h,i,j^** **(0.10–0.26)**	0.03 (0.01–0.08)	0.04 (0.02–0.12)	0.06 (0.03–0.09)	0.05 (0.02–0.08)	0.08 (0.02–0.16)

^a^ A vs. NI, *p* = 0.030; ^b^ T vs. Azoo, *p* = 0.040; ^c^ T vs. A, *p* = 0.001; ^d^ T vs. Azoo, *p* < 0.001; ^e^ A vs. NI, *p* = 0.002; ^f^ A vs. AT, *p* = 0.005; ^g^ A vs. OAT, *p* = 0.002; ^h^ Azoo vs. NI, *p* = 0.001; ^i^ Azoo vs. AT, *p* = 0.002; ^j^ Azoo vs. OAT, *p* = 0.006. The values are presented as medians and interquartile ranges (Q1–Q3). The Kruskal–Wallis ANOVA followed by Dunn’s test was used to assess the differences between groups. A two-tailed *p*-value of <0.05 was considered significant. 6-keto-PGF_1α_—6-keto-Prostaglandin F_1α_, A—Asthenozoospermic group, AT—Asthenoteratozoospermic group, Azoo—Azoospermic group, F—Fertile group, GPX_1_—Glutathione Peroxidase 1, NI—Normozoospermic infertile group, NOS_1_—Nitric Oxide Synthase 1, OAT—Oligoasthenoteratozoospermic group, OT—Oligoteratozoospermic group, PGE_2_—Prostaglandin E_2_, SOD_1_—Superoxide Dismutase 1, T—Teratozoospermic group, n—number of participants. Values with statistical significance are indicated in bold font.

**Table 5:** Correlations between investigated parameters among fertile and infertile men.

**Correlated Parameters**	**Fertile**	
	**R**	***p***
F_2_-isoprostane vs. sperm viability	0.46	0.030
GPX_1_ vs. sperm concentration	0.50	0.023
GPX_1_ vs. concentration of morphologically abnormal sperm	0.50	0.021
PGE_2_ vs. ALA *	–0.58	0.005
6-keto-PGF_1α_ vs. EPA *	0.44	0.040
PGE_2_/6-keto-PGF_1α_ vs. ALA *	–0.48	0.024
PGE_2_/6-keto-PGF_1α_ vs. EPA *	–0.50	0.018
F_2_-isoprostane vs. SOD_1_	0.47	0.029
NOS_1_ vs. SOD_1_	0.49	0.022
**Correlated parameters**	**Infertile**	
	**R**	***p***
PGE_2_/6-keto-PGF_1α_ vs. progressive motility	–0.22	< 0.001
PGE_2_ vs. 6-keto-PGF_1α_	0.28	< 0.001
NOS_1_ vs. SOD_1_	0.35	< 0.001

Spearman’s rank test was used to assess the correlations between analyzed parameters, and a *p*-value of less than 0.05 was considered significant. 6-keto-PGF_1α_—6-keto-Prostaglandin F_1α_, ALA—α-linolenic acid, EPA—eicosapentaenoic acid, GPX_1_—Glutathione Peroxidase 1, NOS_1_—Nitric Oxide Synthase 1, PGE_2_—Prostaglandin E_2_, SOD_1_—Superoxide Dismutase 1, R—Spearman’s rank coefficient. *—PUFA concentrations were determined in our previous study [44].

## Data Availability

The original contributions presented in this study are included in the article/Supplementary Material.

## Supplementary Materials

The following supporting information can be downloaded at: https://www.mdpi.com/article/10.3390/antiox14121470/s1, Table S1: Correlations between examined parameters and standard semen parameters in the group of infertile men; Table S2: Correlations between examined parameters and PUFA concentrations in seminal plasma in the group of infertile men; Table S3: Correlations between examined parameters in the group of infertile men; Table S4: Correlations between examined parameters and standard semen parameters in the group of fertile men; Table S5: Correlations between examined parameters and PUFA concentrations in seminal plasma in the group of fertile men; Table S6: Correlations between examined parameters in the group of fertile men; Figure S1: Comparison of concentrations of examined parameters between groups of infertile men using box plots.; Figure S2: Comparison of ratios of examined parameters between groups of infertile men using box plots.

## Abbreviations

The following abbreviations are used in this manuscript:

6-keto-PGF_1α_	6-keto-prostaglandin F_1α_
AA	Arachidonic acid
ALA	α-Linolenic acid
AT	Asthenoteratozoospermic
A	Asthenozoospermic
Azoo	Azoospermic
BMI	Body Mass Index
COX-1/COX-2	Cyclooxygenase 1/2
CYP 450	Cytochrome P450
DHA	Docosahexaenoic acid
EPA	Eicosapentaenoic acid
F	Fertile
FAMEs	Fatty acid methyl esters
GC-MS/MS	Gas chromatography-tandem mass spectrometry
GLA	γ-Linolenic acid
GPX	Glutathione peroxidase
IsoPs	Isoprostanes
LA	Linoleic acid
NI	Infertile normozoospermic
NO	Nitric oxide
NOS	Nitric oxide synthase
O	Oligozoospermic
OA	Oligoasthenozoospermic
OAT	Oligoasthenoteratozoospermic
OT	Oligoteratozoospermic
OS	Oxidative stress
PGE_2_	Prostaglandin E_2_
PGI_2_	Prostacyclin
PUFAs	Polyunsaturated fatty acids
SOD	Superoxide dismutase
T	Teratozoospermic
WHO	World Health Organization
ROS	Reactive oxygen species

## References

[1] WHO (2021). Laboratory Manual for the Examination and Processing of Human Semen.

[2] WHO (2023). Infertility Prevalence Estimates, 1990–2021.

[3] Leslie S.W., Soon-Sutton T.L., Khan M.A. (2024). Male Infertility. StatPearls.

[4] Boitrelle F., Shah R., Saleh R., Henkel R., Kandil H., Chung E., Vogiatzi P., Zini A., Arafa M., Agarwal A. (2021). The Sixth Edition of the WHO Manual for Human Semen Analysis: A Critical Review and SWOT Analysis. Life.

[5] Boeri L., Kandil H., Ramsay J. (2024). Idiopathic Male Infertility—What Are We Missing?. Arab J. Urol..

[6] Aitken R.J., Smith T.B., Jobling M.S., Baker M.A., De Iuliis G.N. (2014). Oxidative Stress and Male Reproductive Health. Asian J. Androl..

[7] Sharma R.K., Agarwal A. (1996). Role of Reactive Oxygen Species in Male Infertility. Urology.

[8] Sanocka D., Kurpisz M. (2004). Reactive Oxygen Species and Sperm Cells. Reprod. Biol. Endocrinol. Reprod. Biol. Endocrinol..

[9] Kowalczyk A. (2022). The Role of the Natural Antioxidant Mechanism in Sperm Cells. Reprod. Sci..

[10] O’Flaherty C., Scarlata E. (2022). OXIDATIVE STRESS AND REPRODUCTIVE FUNCTION: The Protection of Mammalian Spermatozoa against Oxidative Stress. Reproduction.

[11] Agarwal A., Virk G., Ong C., du Plessis S.S. (2014). Effect of Oxidative Stress on Male Reproduction. World J. Mens Health.

[12] Rodak K., Kratz E.M. (2023). PUFAs and Their Derivatives as Emerging Players in Diagnostics and Treatment of Male Fertility Disorders. Pharmaceuticals.

[13] Aitken R.J. (2017). Reactive Oxygen Species as Mediators of Sperm Capacitation and Pathological Damage. Mol. Reprod. Dev..

[14] Lamirande E.D., Gagnon C. (1993). A Positive Role for the Superoxide Anion in Triggering Hyperactivation and Capacitation of Human Spermatozoa. Int. J. Androl..

[15] Potts R.J., Notarianni L.J., Jefferies T.M. (2000). Seminal Plasma Reduces Exogenous Oxidative Damage to Human Sperm, Determined by the Measurement of DNA Strand Breaks and Lipid Peroxidation. Mutat. Res..

[16] Cannarella R., Crafa A., Barbagallo F., Mongioì L.M., Condorelli R.A., Aversa A., Calogero A.E., La Vignera S. (2020). Seminal Plasma Proteomic Biomarkers of Oxidative Stress. Int. J. Mol. Sci..

[17] Flesch F.M., Gadella B.M. (2000). Dynamics of the Mammalian Sperm Plasma Membrane in the Process of Fertilization. Biochim. Biophys. Acta BBA—Rev. Biomembr..

[18] Lenzi A., Picardo M., Gandini L., Dondero F. (1996). Lipids of the Sperm Plasma Membrane: From Polyunsaturated Fatty Acids Considered as Markers of Sperm Function to Possible Scavenger Therapy. Hum. Reprod. Update.

[19] Collodel G., Castellini C., Lee J.C.-Y., Signorini C. (2020). Relevance of Fatty Acids to Sperm Maturation and Quality. Oxid. Med. Cell Longev..

[20] Safarinejad M.R., Hosseini S.Y., Dadkhah F., Asgari M.A. (2010). Relationship of Omega-3 and Omega-6 Fatty Acids with Semen Characteristics, and Anti-Oxidant Status of Seminal Plasma: A Comparison between Fertile and Infertile Men. Clin. Nutr..

[21] Dyall S.C., Balas L., Bazan N.G., Brenna J.T., Chiang N., da Costa Souza F., Dalli J., Durand T., Galano J.-M., Lein P.J. (2022). Polyunsaturated Fatty Acids and Fatty Acid-Derived Lipid Mediators: Recent Advances in the Understanding of Their Biosynthesis, Structures, and Functions. Prog. Lipid Res..

[22] Moustakli E., Zikopoulos A., Skentou C., Stavros S., Sofikitis N., Georgiou I., Zachariou A. (2024). Integrative Assessment of Seminal Plasma Biomarkers: A Narrative Review Bridging the Gap between Infertility Research and Clinical Practice. J. Clin. Med..

[23] Bieniek J.M., Drabovich A.P., Lo K.C. (2016). Seminal Biomarkers for the Evaluation of Male Infertility. Asian J. Androl..

[24] Kumar N., Singh N.K. (2020). Emerging Role of Novel Seminal Plasma Bio-Markers in Male Infertility: A Review. Eur. J. Obstet. Gynecol. Reprod. Biol..

[25] Drabovich A.P., Saraon P., Jarvi K., Diamandis E.P. (2014). Seminal Plasma as a Diagnostic Fluid for Male Reproductive System Disorders. Nat. Rev. Urol..

[26] Preianò M., Correnti S., Butt T.A., Viglietto G., Savino R., Terracciano R. (2023). Mass Spectrometry-Based Untargeted Approaches to Reveal Diagnostic Signatures of Male Infertility in Seminal Plasma: A New Laboratory Perspective for the Clinical Management of Infertility?. Int. J. Mol. Sci..

[27] Cummins J.M., Jequier A.M., Kan R. (1994). Molecular Biology of Human Male Infertility: Links with Aging, Mitochondrial Genetics, and Oxidative Stress?. Mol. Reprod. Dev..

[28] Hsieh Y.-Y., Chang C.-C., Lin C.-S. (2006). Seminal Malondialdehyde Concentration but Not Glutathione Peroxidase Activity Is Negatively Correlated with Seminal Concentration and Motility. Int. J. Biol. Sci..

[29] Yan L., Liu J., Wu S., Zhang S., Ji G., Gu A. (2014). Seminal Superoxide Dismutase Activity and Its Relationship with Semen Quality and SOD Gene Polymorphism. J. Assist. Reprod. Genet..

[30] Otasevic V., Kalezic A., Macanovic B., Jankovic A., Stancic A., Garalejic E., Korac A., Korac B. (2019). Evaluation of the Antioxidative Enzymes in the Seminal Plasma of Infertile Men: Contribution to Classic Semen Quality Analysis. Syst. Biol. Reprod. Med..

[31] Tramer F., Caponecchia L., Sgrò P., Martinelli M., Sandri G., Panfili E., Lenzi A., Gandini L. (2004). Native Specific Activity of Glutathione Peroxidase (GPx-1), Phospholipid Hydroperoxide Glutathione Peroxidase (PHGPx) and Glutathione Reductase (GR) Does Not Differ between Normo- and Hypomotile Human Sperm Samples. Int. J. Androl..

[32] Zhao Y., Zhao E., Zhang C., Zhang H. (2015). Study of the Changes of Acrosomal Enzyme, Nitric Oxide Synthase, and Superoxide Dismutase of Infertile Patients with Positive Antisperm Antibody in Seminal Plasma. Cell Biochem. Biophys..

[33] Zini A., O’Bryan M.K., Schlegel P.N. (2001). Nitric Oxide Synthase Activity in Human Seminal Plasma. Urology.

[34] Eleutherio E.C.A., Silva Magalhães R.S., de Araújo Brasil A., Monteiro Neto J.R., de Holanda Paranhos L. (2021). SOD1, More than Just an Antioxidant. Arch. Biochem. Biophys..

[35] Peeker R., Abramsson L., Marklund S.L. (1997). Superoxide Dismutase Isoenzymes in Human Seminal Plasma and Spermatozoa. Mol. Hum. Reprod..

[36] Atig F., Raffa M., Ali H.B., Abdelhamid K., Saad A., Ajina M. (2011). Altered Antioxidant Status and Increased Lipid Per-Oxidation in Seminal Plasma of Tunisian Infertile Men. Int. J. Biol. Sci..

[37] Handy D.E., Loscalzo J. (2022). The Role of Glutathione Peroxidase-1 in Health and Disease. Free Radic. Biol. Med..

[38] Tavilani H., Goodarzi M.T., Doosti M., Vaisi-Raygani A., Hassanzadeh T., Salimi S., Joshaghani H.R. (2008). Relationship between Seminal Antioxidant Enzymes and the Phospholipid and Fatty Acid Composition of Spermatozoa. Reprod. Biomed. Online.

[39] Luo Y., Zhu Y., Basang W., Wang X., Li C., Zhou X. (2021). Roles of Nitric Oxide in the Regulation of Reproduction: A Review. Front. Endocrinol..

[40] Huang C., Cao X., Pang D., Li C., Luo Q., Zou Y., Feng B., Li L., Cheng A., Chen Z. (2018). Is Male Infertility Associated with Increased Oxidative Stress in Seminal Plasma? A-Meta Analysis. Oncotarget.

[41] Davidoff M.S., Middendorff R., Mayer B., Holstein A.F. (1995). Nitric Oxide Synthase (NOS-I) in Leydig Cells of the Human Testis. Arch. Histol. Cytol..

[42] Lewis S.E.M., Donnelly E.T., Sterling E.S.L., Kennedy M.S., Thompson W., Chakravarthy U. (1996). Nitric Oxide Synthase and Nitrite Production in Human Spermatozoa: Evidence That Endogenous Nitric Oxide Is Beneficial to Sperm Motility. Mol. Hum. Repod..

[43] Garrido N., Meseguer M., Alvarez J., Simón C., Pellicer A., Remohí J. (2004). Relationship among Standard Semen Parameters, Glutathione Peroxidase/Glutathione Reductase Activity, and mRNA Expression and Reduced Glutathione Content in Ejaculated Spermatozoa from Fertile and Infertile Men. Fertil. Steril..

[44] Rodak K., Grajzer M., Kokot I., Faundez R., Gilowska I., Prescha A., Kratz E.M. (2025). GC–MS/MS Analysis of Seminal Plasma PUFAs in Distinct Subgroups of Infertile Men: Diagnostic Potential and Insight into Mechanisms of Male Infertility. Sci. Rep..

[45] Wang C., Swerdloff R.S. (2014). Limitations of Semen Analysis as a Test of Male Fertility and Anticipated Needs from Newer Tests. Fertil. Steril..

[46] Fraczek M., Szkutnik D., Sanocka D., Kurpisz M. (2001). [Peroxidation components of sperm lipid membranes in male infertility]. Ginekol. Pol..

[47] Seshadri S., Bates M., Vince G., Jones D.I.L. (2009). ORIGINAL ARTICLE: The Role of Cytokine Expression in Different Subgroups of Subfertile Men. Am. J. Reprod. Immunol..

[48] Iwasaki A., Gagnon C. (1992). Formation of Reactive Oxygen Species in Spermatozoa of Infertile Patients*. Fertil. Steril..

[49] Zini A., de Lamirande E., Gagnon C. (1993). Reactive Oxygen Species in Semen of Infertile Patients: Levels of Superoxide Dismutase- and Catalase-like Activities in Seminal Plasma and Spermatozoa. Int. J. Androl..

[50] Capra V., Rovati G.E., Mangano P., Buccellati C., Murphy R.C., Sala A. (2015). Transcellular Biosynthesis of Eicosanoid Lipid Mediators. Biochim. Biophys. Acta BBA—Mol. Cell Biol. Lipids.

[51] Frungieri M.B., Gonzalez-Calvar S.I., Matzkin M.E., Mayerhofer A., Calandra R.S. (2007). Sources and Functions of Prostaglandins in the Testis: Evidence for Their Relevance in Male (in)Fertility. Anim. Reprod..

[52] Templeton A.A., Cooper I., Kelly R.W. (1978). Prostaglandin Concentrations in the Semen of Fertile Men. Reproduction.

[53] Bygdeman M., Fredricsson B., Svanborg K., Samuelsson B. (1970). The Relation between Fertility and Prostaglandin Content of Seminal Fluid in Man. Fertil. Steril..

[54] Collier J.G., Flower R.J., Stanton S.L. (1975). Seminal Prostaglandins in Infertile Men. Fertil. Steril..

[55] Bendvold E., Svanborg K., Eneroth P., Gottlieb C., Bygdeman M. (1984). The Natural Variations in Prostaglandin Concentration in Human Seminal Fluid and Its Relation to Sperm Quality*. Fertil. Steril..

[56] Kelly R.W., Cooper I., Templeton A.A. (1979). Reduced Prostaglandin Levels in the Semen of Men with Very High Sperm Concentrations. Reproduction.

[57] Isidori A., Conte D., Laguzzi G., Giovenco P., Dondero F. (1980). Role of Seminal Prostaglandins in Male Fertility. I. Relationship of Prostaglandin E and 19-OH Prostaglandin E with Seminal Parameters. J. Endocrinol. Invest..

[58] Holtel M., Chosed R.J., Zimmerman S., Wynia B., Roudebush W.E. (2019). Relationship between Seminal Prostaglandins on Intrauterine Insemination Pregnancy Outcomes. Fertil. Steril..

[59] Samuelsson B. (1963). Isolation and Identification of Prostaglandins from Human Seminal Plasma: 18. PROSTAGLANDINS AND RELATED FACTORS. J. Biol. Chem..

[60] Jonsson H.T., Middleditch B.S., Desiderio D.M. (1975). Prostaglandins in Human Seminal Fluid: Two Novel Compounds. Science.

[61] Hoxha M., Barbonetti A., Zappacosta B. (2023). Arachidonic Acid Pathways and Male Fertility: A Systematic Review. Int. J. Mol. Sci..

[62] Lewy R.I., Bills T.K., Dalton J., Smith J.B., Silver M.J. (1979). 19-Hydroxy-Prostaglandin E and Infertility in Human Males. Prostaglandins Med..

[63] Amor H., Dahadhah F.W., Jankowski P.M., Al Nasser R., Jung L., Juhasz-Böss I., Solomayer E.F., Hammadeh M.E. (2025). Relationship Between Prostaglandin and Interleukin Concentrations in Seminal Fluid and Their Influence on the Rate of Fertilization in Men Undergoing ICSI. Int. J. Mol. Sci..

[64] Cao H., Xiao L., Park G., Wang X., Azim A.C., Christman J.W., van Breemen R.B. (2008). An Improved LC-MS-MS Method for the Quantification of Prostaglandins E2 and D2 Production in Biological Fluids. Anal. Biochem..

[65] Schlegel W., Meyer J. (1986). Seminal Prostaglandins in Men with Subnormal Sperm Motility and Therapeutic Treatment. Prostaglandins.

[66] Huleihel M., Lunenfeld E., Horowitz S., Levy A., Potashnik G., Mazor M., Glezerman M. (1999). Expression of IL-12, IL-10, PGE2, sIL-2R and sIL-6R in Seminal Plasma of Fertile and Infertile Men. Andrologia.

[67] Chen X., Wu B., Shen X., Wang X., Ping P., Miao M., Liang N., Yin H., Shi H., Qian J. (2023). Relevance of PUFA-Derived Metabolites in Seminal Plasma to Male Infertility. Front. Endocrinol..

[68] Zhang Y., Peng Y., Wang Y., Xu J., Yan H. (2024). Novel Underlying Genetic Markers for Asthenozoospermia Due to Abnormal Spermatogenesis and Reproductive Organ Inflammation. Exp. Ther. Med..

[69] Hagan S., Khurana N., Chandra S., Abdel-Mageed A.B., Mondal D., Hellstrom W.J.G., Sikka S.C. (2015). Differential Expression of Novel Biomarkers (TLR-2, TLR-4, COX-2, and Nrf-2) of Inflammation and Oxidative Stress in Semen of Leukocytospermia Patients. Andrology.

[70] Salvolini E., Lucarini G., Vignini A., Buldreghini E., Mazzanti L., Primio R.D., Balercia G. (2014). Involvement of Interleukin-1β, Cyclooxygenase-2, and Hypoxia-Inducible Factor -1α in Idiopathic Asthenozoospermia. Ital. J. Anat. Embryol..

[71] Bendvold E., Svanborg K., Bygdeman M., Norén S. (1985). On the Origin of Prostaglandins in Human Seminal Fluid. Int. J. Androl..

[72] Vigor C., Bertrand-Michel J., Pinot E., Oger C., Vercauteren J., Le Faouder P., Galano J.-M., Lee J.C.-Y., Durand T. (2014). Non-Enzymatic Lipid Oxidation Products in Biological Systems: Assessment of the Metabolites from Polyunsaturated Fatty Acids. J. Chromatogr. B.

[73] Morrow J.D., Awad J.A., Boss H.J., Blair I.A., Roberts L.J. (1992). Non-Cyclooxygenase-Derived Prostanoids (F2-Isoprostanes) Are Formed in Situ on Phospholipids. Proc. Natl. Acad. Sci. USA.

[74] Montuschi P., Barnes P.J., Roberts L.J. (2004). Isoprostanes: Markers and Mediators of Oxidative Stress. FASEB J..

[75] Milne G.L., Dai Q., Roberts L.J. (2015). The Isoprostanes--25 Years Later. Biochim. Biophys. Acta.

[76] Roberts L.J., Morrow J.D. (2002). Products of the Isoprostane Pathway: Unique Bioactive Compounds and Markers of Lipid Peroxidation. Cell Mol. Life Sci..

[77] Collodel G., Moretti E., Longini M., Pascarelli N.A., Signorini C. (2018). Increased F2-Isoprostane Levels in Semen and Immunolocalization of the 8-Iso Prostaglandin F2α in Spermatozoa from Infertile Patients with Varicocele. Oxid. Med. Cell Longev..

[78] Collodel G., Castellini C., Iacoponi F., Noto D., Signorini C. (2020). Cytosolic Phospholipase A2 and F2 Isoprostanes Are Involved in Semen Quality and Human Infertility-A Study on Leucocytospermia, Varicocele and Idiopathic Infertility. Andrologia.

[79] Moretti E., Signorini C., Ferretti F., Noto D., Collodel G. (2022). A Study to Validate the Relevance of Semen F2-Isoprostanes on Human Male Infertility. Int. J. Environ. Res. Public. Health.

[80] Longini M., Moretti E., Signorini C., Noto D., Iacoponi F., Collodel G. (2020). Relevance of Seminal F2-Dihomo-IsoPs, F2-IsoPs and F4-NeuroPs in Idiopathic Infertility and Varicocele. Prostaglandins Other Lipid Mediat..

[81] Moretti E., Signorini C., Menchiari S., Liguori L., Corsaro R., Gambera L., Collodel G. (2025). Are F2-Isoprostanes a Better Marker of Semen Lipid Peroxidation than MDA in Reproductive Pathologies with Inflammatory Basis?. Cytokine.

[82] Yang H.-Y., Lee T.-H. (2015). Antioxidant Enzymes as Redox-Based Biomarkers: A Brief Review. BMB Rep..

[83] Chung J., Ha J.W., Park Y.-B., Lee S.-W. (2025). Serum Glutathione Peroxidase-3 Concentration at Diagnosis as a Biomarker for Assessing Disease Activity and Damage of Antineutrophil Cytoplasmic Antibody-Associated Vasculitis at Diagnosis. Front. Mol. Biosci..

[84] Holthoff J.H., Harville Y., Herzog C., Juncos L.A., Karakala N., Arthur J.M. (2023). SOD1 Is a Novel Prognostic Biomarker of Acute Kidney Injury Following Cardiothoracic Surgery. BMC Nephrol..

[85] Bezna M.C., Istratoaie O., Danoiu S., Bezna M., Toader D.M., Mustafa R.E., Donoiu I., Pisoschi C., Melinte P.R. (2023). GPx-1 Isoenzyme—Biomarker in the Assessment of Lipid Oxidative Risk, Immunogenesis and Arrhythmogenic Potential. Eur. Heart J..

[86] Lei J., Paules C., Nigrini E., Rosenzweig J.M., Bahabry R., Farzin A., Yang S., Northington F.J., Oros D., McKenney S. (2017). Umbilical Cord Blood NOS1 as a Potential Biomarker of Neonatal Encephalopathy. Front. Pediatr..

[87] Król-Kulikowska M., Banasik M., Kepinska M. (2024). The Effect of Selected Nitric Oxide Synthase Polymorphisms on the Risk of Developing Diabetic Nephropathy. Antioxidants.

[88] Bortolin R.C., Gasparotto J., Vargas A.R., da Silva Morrone M., Kunzler A., Henkin B.S., Chaves P.R., Roncato S., Gelain D.P., Moreira J.C.F. (2017). Effects of Freeze-Thaw and Storage on Enzymatic Activities, Protein Oxidative Damage, and Immunocontent of the Blood, Liver, and Brain of Rats. Biopreservation Biobanking.

[89] Murias M., Rachtan M., Jodynis-Liebert J. (2005). Effect of Multiple Freeze–Thaw Cycles of Cytoplasm Samples on the Activity of Antioxidant Enzymes. J. Pharmacol. Toxicol. Methods.

[90] Yin T., Yue X., Li Q., Zhou X., Dong R., Chen J., Zhang R., Wang X., He S., Jiang T. (2024). The Association Between the Levels of Oxidative Stress Indicators (MDA, SOD, and GSH) in Seminal Plasma and the Risk of Idiopathic Oligo-Asthenotera-Tozoospermia: Does Cu or Se Level Alter the Association?. Biol. Trace Elem. Res..

[91] Wdowiak A., Bakalczuk S., Bakalczuk G. (2015). Decreased Activity of Superoxide Dismutase in the Seminal Plasma of Infertile Men Correlates with Increased Sperm Deoxyribonucleic Acid Fragmentation during the First Hours after Sperm Donation. Andrology.

[92] Murawski M., Saczko J., Marcinkowska A., Chwiłkowska A., Gryboś M., Banaś T. (2007). Evaluation of Superoxide Dismutase Activity and Its Impact on Semen Quality Parameters of Infertile Men. Folia Histochem. Cytobiol..

[93] Marzec-Wróblewska U., Kamiński P., Łakota P., Szymański M., Wasilow K., Ludwikowski G., Kuligowska-Prusińska M., Odrowąż-Sypniewska G., Stuczyński T., Michałkiewicz J. (2011). Zinc and Iron Concentration and SOD Activity in Human Semen and Seminal Plasma. Biol. Trace Elem. Res..

[94] Alkan I., Simşek F., Haklar G., Kervancioğlu E., Ozveri H., Yalçin S., Akdaş A. (1997). Reactive Oxygen Species Production by the Spermatozoa of Patients with Idiopathic Infertility: Relationship to Seminal Plasma Antioxidants. J. Urol..

[95] Khosrowbeygi A., Zarghami N. (2007). Levels of Oxidative Stress Biomarkers in Seminal Plasma and Their Relationship with Seminal Parameters. BMC Clin. Pathol..

[96] Zini A., Garrels K., Phang D. (2000). Antioxidant Activity in the Semen of Fertile and Infertile Men. Urology.

[97] Dandekar S.P., Nadkarni G.D., Kulkarni V.S., Punekar S. (2002). Lipid Peroxidation and Antioxidant Enzymes in Male Infertility. J. Postgrad. Med..

[98] Giannattasio A., De Rosa M., Smeraglia R., Zarrilli S., Cimmino A., Di Rosario B., Ruggiero R., Colao A., Lombardi G. (2002). Glutathione Peroxidase (GPX) Activity in Seminal Plasma of Healthy and Infertile Males. J. Endocrinol. Invest..

[99] Ozbek E., Turkoz Y., Gokdeniz R., Davarci M., Ozugurlu F. (2000). Increased Nitric Oxide Production in the Spermatic Vein of Patients with Varicocele. Eur. Urol..

[100] Hsieh Y., Sun Y., Chang C., Lee Y., Tsai H., Lin C. (2002). Superoxide Dismutase Activities of Spermatozoa and Seminal Plasma Are Not Correlated with Male Infertility. J. Clin. Lab. Anal..

[101] Miesel R., Jedrzejczak P., Sanocka D., Kurpisz M.K. (1997). Severe Antioxidase Deficiency in Human Semen Samples with Pathological Spermiogram Parameters. Andrologia.

[102] Sanocka D., Miesel R., Jedrzejczak P., Chełmonska-Soyta A.C., Kurpisz M. (1997). Effect of Reactive Oxygen Species and the Activity of Antioxidant Systems on Human Semen; Association with Male Infertility. Int. J. Androl..

[103] Chyra-Jach D., Kaletka Z., Dobrakowski M., Machoń-Grecka A., Kasperczyk S., Birkner E., Kasperczyk A. (2018). The Associations between Infertility and Antioxidants, Proinflammatory Cytokines, and Chemokines. Oxid. Med. Cell Longev..

[104] Tamilselvan K., Ravinder S., Thangavel G., Subashini A.S., Ramaswamy P. (2015). A Study of Variation in the Levels of Seminal Plasma Superoxide Dismutase and Glutathione Peroxidase in Normospermic and Oligozoospermic Men. Int. J. Biomed. Res..

[105] Tkaczuk-Włach J., Kankofer M., Jakiel G. (2002). Activity of Superoxide Dismutase and Glutathione Peroxidase in Human Semen in Normozoospermia and Spermatopathy. Ann. Univ. Mariae Curie Sklodowska Med..

[106] Zelen I., Mitrović M., Jurisic-Skevin A., Arsenijević S. (2010). Activity of Superoxide Dismutase and Catalase and Content of Malondialdehyde in Seminal Plasma of Infertile Patients. Med. Pregl..

[107] Ramya T., Misro M.M., Sinha D., Nandan D., Mithal S. (2011). Altered Levels of Seminal Nitric Oxide, Nitric Oxide Synthase, and Enzymatic Antioxidants and Their Association with Sperm Function in Infertile Subjects. Fertil. Steril..

[108] Ammar O., Haouas Z., Hamouda B., Hamdi H., Hellara I., Jlali A., Cheikh H.B., Mehdi M. (2019). Relationship between Sperm DNA Damage with Sperm Parameters, Oxidative Markers in Teratozoospermic Men. Eur. J. Obstet. Gynecol. Reprod. Biol..

[109] Al-Maliki R.S. (2024). Elevated Seminal Plasma TLR-2 Levels Are Associated with Leukocytospermia. Dubai Med. J..

[110] Crisol L., Matorras R., Aspichueta F., Expósito A., Hernández M.L., Ruiz-Larrea M.B., Mendoza R., Ruiz-Sanz J.I. (2012). Glutathione Peroxidase Activity in Seminal Plasma and Its Relationship to Classical Sperm Parameters and in Vitro Fertilization-Intracytoplasmic Sperm Injection Outcome. Fertil. Steril..

[111] Safarinejad M.R. (2011). Effect of Omega-3 Polyunsaturated Fatty Acid Supplementation on Semen Profile and Enzymatic Anti-Oxidant Capacity of Seminal Plasma in Infertile Men with Idiopathic Oligoasthenoteratospermia: A Double-Blind, Placebo-Controlled, Randomised Study. Andrologia.

[112] Nartey M.N.N., Shimizu H., Sugiyama H., Higa M., Syeda P.K., Nishimura K., Jisaka M., Yokota K. (2023). Eicosapentaenoic Acid Induces the Inhibition of Adipogenesis by Reducing the Effect of PPARγ Activator and Mediating PKA Activation and Increased COX-2 Expression in 3T3-L1 Cells at the Differentiation Stage. Life.

[113] Eibl G. (2008). The Role of PPAR-γ and Its Interaction with COX-2 in Pancreatic Cancer. PPAR Res..

[114] Tunc O., Bakos H.W., Tremellen K. (2011). Impact of Body Mass Index on Seminal Oxidative Stress. Andrologia.

[115] Jing J., Peng Y., Fan W., Han S., Peng Q., Xue C., Qin X., Liu Y., Ding Z. (2023). Obesity-induced Oxidative Stress and Mitochondrial Dysfunction Negatively Affect Sperm Quality. FEBS Open Bio..

[116] Saleh R.A., Agarwal A., Sharma R.K., Nelson D.R., Thomas A.J. (2002). Effect of Cigarette Smoking on Levels of Seminal Oxidative Stress in Infertile Men: A Prospective Study. Fertil. Steril..

[117] Ou Z., Wen Q., Deng Y., Yu Y., Chen Z., Sun L. (2020). Cigarette Smoking Is Associated with High Level of Ferroptosis in Seminal Plasma and Affects Semen Quality. Reprod. Biol. Endocrinol..

[118] Ragheb A.M., Sabanegh E.S. (2009). Smoking and Male Fertility: A Contemporary Review. Arch. Med. Sci. Spec. Issues.

[119] Yousefniapasha Y., Jorsaraei G., Gholinezhadchari M., Mahjoub S., Hajiahmadi M., Farsi M. (2015). Nitric Oxide Levels and Total Antioxidant Capacity in The Seminal Plasma of Infertile Smoking Men. Cell J. Yakhteh.

[120] Viloria T., Meseguer M., Martínez-Conejero J.A., O’Connor J.E., Remohí J., Pellicer A., Garrido N. (2010). Cigarette Smoking Affects Specific Sperm Oxidative Defenses but Does Not Cause Oxidative DNA Damage in Infertile Men. Fertil. Steril..

[121] Madej D., Granda D., Sicinska E., Kaluza J. (2021). Influence of Fruit and Vegetable Consumption on Antioxidant Status and Semen Quality: A Cross-Sectional Study in Adult Men. Front. Nutr..

[122] Bouhadana D., Godin Pagé M.-H., Montjean D., Bélanger M.-C., Benkhalifa M., Miron P., Petrella F. (2025). The Role of Antioxidants in Male Fertility: A Comprehensive Review of Mechanisms and Clinical Applications. Antioxidants.

[123] Strzeżek J., Fraser L., Kuklińska M., Dziekońska A., Lecewicz M. (2004). Effects of Dietary Supplementation with Polyunsaturated Fatty Acids and Antioxidants on Biochemical Characteristics of Boar Semen. Reprod. Biol..

