# Supplementary material for: Biomarkers and Mechanisms of Male Infertility: Evaluation of Antioxidant Enzymes and Arachidonic Acid Derivatives in Seminal Plasma from Fertile and Infertile Men
Source: Antioxidants (Basel). 2025 Dec 7;14(12):1470. doi: 10.3390/antiox14121470 (PMC12729684; doi:10.3390/antiox14121470)
Supplement: Supplementary file 1 [file antioxidants-14-01470-s001.zip › antioxidants-4001537-supplementary.pdf]

Table S1. Correlations between examined parameters and standard semen parameters in the group of infertile men.

Parameters	Sperm concentration [×10⁶/mL]		Sperm count per ejaculate [×10⁶]		Sperm viability [%]		Sperm total motility [%]		Sperm progressive motility [%]		Sperm normal morphology [%]		Concentration of morphologically abnormal sperm [×10⁶/mL]	
	**R**	***p***	**R**	***p***	**R**	***p***	**R**	***p***	**R**	***p***	**R**	***p***	**R**	***p***
**PGE_2**	-0,04	0.551	-0,06	0.318	-0,04	0.574	-0,04	0.504	-0,09	0.135	0,04	0.483	0,03	0.625
**6-keto-$PGF_{1α}$**	0.03	0.605	0.09	0.157	-0.04	0.533	0.06	0.383	0.19	0.003	0.08	0.232	0.03	0.587
**F_2-isoprostane**	-0.01	0.913	-0.04	0.554	-0.03	0.686	-0.01	0.842	-0.02	0.798	0.08	0.233	0.05	0.447
**SOD_1**	0.05	0.423	-0.02	0.739	-0.06	0.342	-0.09	0.144	-0.11	0.088	0.03	0.593	0.07	0.270
**GPX_1**	0.11	0.095	0.03	0.611	0.02	0.809	0.01	0.595	-0.04	0.557	0.02	0.710	0.10	0.127
**NOS_1**	0.04	0.524	0.13	0.048	-0.07	0.277	0.01	0.837	0.13	0.042	0.17	0.009	0.13	0.035
**NOS_1/SOD_1**	0.03	0.639	0.13	0.040	-0.07	0.325	0.04	0.512	0.17	0.008	0.08	0.237	0.05	0.441
**NOS_1/GPX_1**	-0.03	0.631	0.08	0.222	-0.07	0.313	0.01	0.979	0.11	0.108	0.09	0.165	0.03	0.631
**GPX_1/SOD_1**	0.03	0.608	0.01	0.986	0.02	0.775	0.03	0.644	0.01	0.884	-0.02	0.734	0.01	0.907
**PGE_2/6-keto-$PGF_{1α}$**	-0.08	0.209	-0.14	0.022	-0.01	0.877	-0.08	0.205	**-0.22**	**< 0.001**	0.01	0.988	0.01	0.919

Spearman's rank test was used to assess the correlations between analyzed parameters, and a *p*-value of < 0.05 was considered significant. Weak, moderate, and higher ($0.20 < |R|$, $p < 0.05$) significant correlations are marked in bold and red. 6-keto-$PGF_{1α}$–6-keto-Prostaglandin $F_{1α}$, GPX_1–Glutathione Peroxidase 1, NOS_1–Nitric Oxide Synthase 1, PGE_2–Prostaglandin E_2, SOD_1 – Superoxide Dismutase 1, R – Spearman's rank coefficient.

Table S2. Correlations between examined parameters and PUFA concentrations in seminal plasma in the group of infertile men.

Parameters	LA		ALA		GLA		AA		EPA		DHA	
	R	*p*	R	*p*	R	*p*	R	*p*	R	*p*	R	*p*
**PGE_2**	-0.03	0.590	0.14	0.032	0.04	0.538	-0.03	0.587	-0.09	0.144	-0.05	0.407
**6-keto-$PGF_{1\alpha}$**	0.02	0.730	0.05	0.390	0.05	0.411	0.02	0.753	0.05	0.463	0.06	0.368
**F_2-isoprostane**	-0.01	0.929	0.09	0.147	-0.02	0.777	-0.05	0.472	0.08	0.182	-0.06	0.359
**SOD_1**	0.04	0.575	-0.03	0.678	-0.05	0.464	0.03	0.666	-0.04	0.495	0.02	0.749
**GPX_1**	0.16	0.019	0.08	0.259	0.17	0.010	0.11	0.086	0.04	0.572	0.16	0.016
**NOS_1**	0.05	0.426	-0.04	0.537	-0.08	0.208	0.05	0.417	0.02	0.750	0.01	0.831
**NOS_1/SOD_1**	0.04	0.523	-0.02	0.798	-0.04	0.563	0.06	0.375	0.08	0.237	0.04	0.523
**NOS_1/GPX_1**	-0.07	0.312	-0.08	0.208	-0.18	0.008	-0.04	0.538	-0.01	0.865	-0.09	0.158
**GPX_1/SOD_1**	0.04	0.582	0.05	0.477	0.12	0.074	0.02	0.728	0.03	0.651	0.07	0.293
**PGE_2/6-keto-$PGF_{1\alpha}$**	-0.09	0.164	0.06	0.382	-0.03	0.665	-0.07	0.260	-0.14	0.025	-0.13	0.046

Spearman's rank test was used to assess the correlations between analyzed parameters, and a *p*-value of < 0.05 was considered significant. 6-keto-$PGF_{1\alpha}$ – 6-keto-Prostaglandin $F_{1\alpha}$, AA – arachidonic acid, ALA – α-linolenic acid, DHA – docosahexaenoic acid, EPA – eicosapentaenoic acid, GLA – γ-linolenic acid, GPX_1 – Glutathione Peroxidase 1, LA – linoleic acid, NOS_1 – Nitric Oxide Synthase 1, PGE_2 – Prostaglandin E_2, SOD_1 – Superoxide Dismutase 1, R – Spearman's rank coefficient.

Table S3. Correlations between examined parameters in the group of infertile men.

Parameters	PGE_2		6-keto-$PGF_{1\alpha}$		F_2-isoprostane		NOS_1		GPX_1		SOD_1	
	**R**	***p***	**R**	***p***	**R**	***p***	**R**	***p***	**R**	***p***	**R**	***p***
**PGE_2**			**0.28**	**< 0.001**	-0.02	0.711	-0.10	0.116	0.08	0.246	-0.15	0.015
**6-keto-$PGF_{1\alpha}$**	**0.28**	**< 0.001**			-0.08	0.223	0.04	0.561	-0.09	0.191	-0.11	0.096
**F_2-isoprostane**	-0.02	0.711	-0.08	0.223			0.06	0.368	0.07	0.283	0.03	0.691
**NOS_1**	-0.10	0.116	0.04	0.561	0.06	0.368			-0.01	0.856	**0.35**	**< 0.001**
**GPX_1**	0.08	0.246	-0.09	0.191	0.07	0.283	-0.01	0.856			0.12	0.078
**SOD_1**	-0.15	0.015	-0.11	0.096	0.03	0.691	**0.35**	**< 0.001**	0.12	0.078		

Spearman's rank test was used to assess the correlations between analyzed parameters, and a *p*-value of < 0.05 was considered significant. Weak, moderate, and higher ($0.20 < |R|$, $p < 0.05$) significant correlations are marked in bold and red. 6-keto-$PGF_{1\alpha}$–6-keto-Prostaglandin $F_{1\alpha}$, GPX_1–Glutathione Peroxidase 1, NOS_1–Nitric Oxide Synthase 1, PGE_2–Prostaglandin E_2, SOD_1 – Superoxide Dismutase 1, R – Spearman's rank coefficient.

Table S4. Correlations between examined parameters and standard semen parameters in the group of fertile men.

Parameters	Sperm concentration [×10⁶/mL]		Sperm count per ejaculate [×10⁶]		Sperm viability [%]		Sperm total motility [%]		Sperm progressive motility [%]		Sperm normal morphology [%]		Concentration of morphologically abnormal sperm [×10⁶/mL]	
	R	*p*	R	*p*	R	*p*	R	*p*	R	*p*	R	*p*	R	*p*
**PGE_2**	0.11	0.636	0.05	0.816	-0.07	0.770	0.16	0.465	0.19	0.399	-0.29	0.186	0.09	0.687
**6-keto-$PGF_{1\alpha}$**	-0.16	0.465	-0.25	0.268	-0.15	0.519	0.01	0.990	-0.04	0.871	0.08	0.720	-0.16	0.487
**F_2-isoprostane**	-0.08	0.711	-0.06	0.778	**0.46**	**0.030**	0.12	0.594	-0.09	0.697	0.01	0.966	-0.11	0.636
**SOD_1**	-0.05	0.809	-0.02	0.938	0.24	0.275	-0.09	0.695	-0.20	0.374	0.20	0.362	-0.03	0.887
**GPX_1**	**0.49**	**0.023**	0.42	0.058	0.27	0.241	0.21	0.351	0.07	0.760	0.12	0.590	**0.50**	**0.021**
**NOS_1**	0.13	0.570	0.14	0.533	0.18	0.420	-0.02	0.915	-0.03	0.901	0.08	0.733	0.15	0.500
**NOS_1/SOD_1**	0.05	0.824	-0.04	0.885	-0.15	0.513	-0.04	0.871	0.09	0.697	-0.11	0.633	0.04	0.867
**NOS_1/GPX_1**	-0.31	0.177	-0.19	0.410	-0.15	0.518	-0.13	0.582	-0.03	0.904	-0.02	0.916	-0.28	0.211
**GPX_1/SOD_1**	0.32	0.153	0.24	0.289	0.05	0.836	0.16	0.486	0.12	0.594	-0.16	0.480	0.29	0.201
**PGE_2/6-keto-$PGF_{1\alpha}$**	0.23	0.308	0.19	0.396	-0.02	0.915	0.13	0.566	0.13	0.553	-0.34	0.122	0.19	0.388

Spearman's rank test was used to assess the correlations between analyzed parameters, and a *p*-value of < 0.05 was considered significant. Weak, moderate, and higher, (0.20 < |R|, p < 0.05) significant correlations are marked in bold and green. 6-keto-$PGF_{1\alpha}$–6-keto-Prostaglandin $F_{1\alpha}$, GPX_1–Glutathione Peroxidase 1, NOS_1–Nitric Oxide Synthase 1, PGE_2–Prostaglandin E_2, SOD_1 – Superoxide Dismutase 1, R – Spearman's rank coefficient.

Table S5. Correlations between examined parameters and PUFA concentrations in seminal plasma in the group of fertile men.

Parameters	LA		ALA		GLA		AA		EPA		DHA	
	**R**	***p***	**R**	***p***	**R**	***p***	**R**	***p***	**R**	***p***	**R**	***p***
**PGE_2**	-0.08	0.736	**-0.58**	**0.004**	-0.27	0.230	-0.09	0.676	-0.36	0.100	0.01	0.962
**6-keto-$PGF_{1\alpha}$**	0.29	0.198	-0.09	0.680	0.27	0.217	0.09	0.699	**0.44**	**0.040**	0.18	0.431
**F_2-isoprostane**	0.10	0.663	0.16	0.476	-0.18	0.416	0.33	0.135	-0.10	0.663	0.36	0.101
**SOD_1**	0.28	0.214	0.41	0.059	0.26	0.246	0.22	0.329	0.18	0.428	0.06	0.797
**GPX_1**	-0.18	0.440	-0.20	0.375	-0.12	0.610	-0.27	0.229	-0.18	0.434	-0.14	0.548
**NOS_1**	0.16	0.477	0.35	0.116	0.15	0.513	0.30	0.179	0.10	0.673	0.15	0.503
**NOS_1/SOD_1**	0.08	0.740	-0.04	0.859	0.02	0.938	0.23	0.296	0.01	0.986	0.19	0.391
**NOS_1/GPX_1**	0.12	0.598	0.27	0.239	0.12	0.618	0.28	0.225	0.18	0.434	0.12	0.618
**GPX_1/SOD_1**	-0.26	0.253	-0.40	0.071	-0.31	0.171	-0.36	0.109	-0.26	0.260	-0.15	0.504
**PGE_2/6-keto-$PGF_{1\alpha}$**	-0.11	0.633	**-0.48**	**0.024**	-0.35	0.106	-0.05	0.809	**-0.50**	**0.018**	0.02	0.934

Spearman's rank test was used to assess the correlations between analyzed parameters, and a *p*-value of < 0.05 was considered significant. Weak, moderate, and higher ($0.20 < |R|$, $p < 0.05$) significant correlations are marked in bold and green. 6-keto-$PGF_{1\alpha}$ – 6-keto-Prostaglandin $F_{1\alpha}$, AA – arachidonic acid, ALA – α-linolenic acid, DHA – docosahexaenoic acid, EPA – eicosapentaenoic acid, GLA – γ-linolenic acid, GPX_1 – Glutathione Peroxidase 1, LA – linoleic acid, NOS_1 – Nitric Oxide Synthase 1, PGE_2 – Prostaglandin E_2, SOD_1 – Superoxide Dismutase 1, R – Spearman's rank coefficient.

Table S6. Correlations between examined parameters in the group of fertile men.

Parameters	PGE_2		6-keto-$PGF_{1\alpha}$		F_2-isoprostane		NOS_1		GPX_1		SOD_1	
	R	*p*	R	*p*	R	*p*	R	*p*	R	*p*	R	*p*
**PGE_2**			-0.02	0.923	-0.18	0.427	-0.25	0.253	-0.08	0.746	-0.26	0.244
**6-keto-$PGF_{1\alpha}$**	-0.02	0.923			0.07	0.772	-0.40	0.068	0.07	0.758	-0.18	0.425
**F_2-isoprostane**	-0.18	0.427	0.07	0.772			0.21	0.359	-0.02	0.929	**0.47**	**0.029**
**NOS_1**	-0.25	0.253	-0.40	0.068	0.21	0.359			-0.28	0.211	**0.49**	**0.022**
**GPX_1**	-0.08	0.746	0.07	0.758	-0.02	0.929	-0.28	0.211			0.07	0.763
**SOD_1**	-0.26	0.244	-0.18	0.425	**0.047**	**0.029**	**0.49**	**0.022**	0.07	0.763		

Spearman's rank test was used to assess the correlations between analyzed parameters, and a *p*-value of < 0.05 was considered significant. Weak, moderate, and higher, ($0.20 < |R|$, $p < 0.05$) significant correlations are marked in bold and red. 6-keto-$PGF_{1\alpha}$–6-keto-Prostaglandin $F_{1\alpha}$, GPX_1–Glutathione Peroxidase 1, NOS_1–Nitric Oxide Synthase 1, PGE_2–Prostaglandin E_2, SOD_1 – Superoxide Dismutase 1, R – Spearman's rank coefficient.

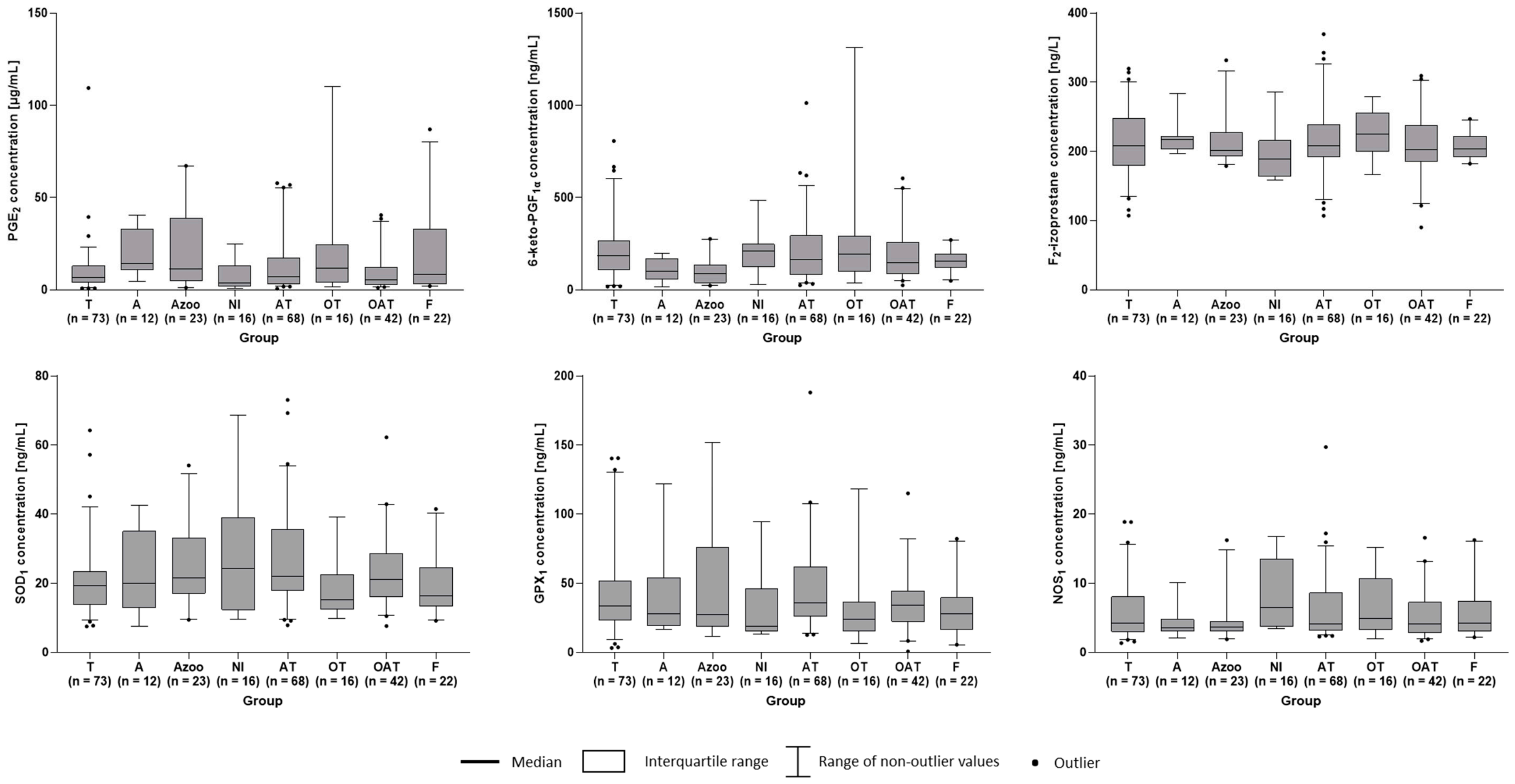

Figure S1. Comparison of concentrations of examined parameters between groups of infertile men using box plots. 6-keto-PGF$_{1\alpha}$ – 6-keto-Prostaglandin F$_{1\alpha}$, A – Asthenozoospermic group, AT – Asthenoteratozoospermic group, Azoo – Azoospermic group, F – Fertile group, GPX$_1$ – Glutathione Peroxidase 1, NI – Normozoospermic infertile group, NOS$_1$ – Nitric Oxide Synthase 1, OAT – Oligoasthenoteratozoospermic group, OT – Oligoteratozoospermic group, PGE$_2$ – Prostaglandin E$_2$, SOD$_1$ – Superoxide Dismutase 1, T – Teratozoospermic group, n – number of participants.

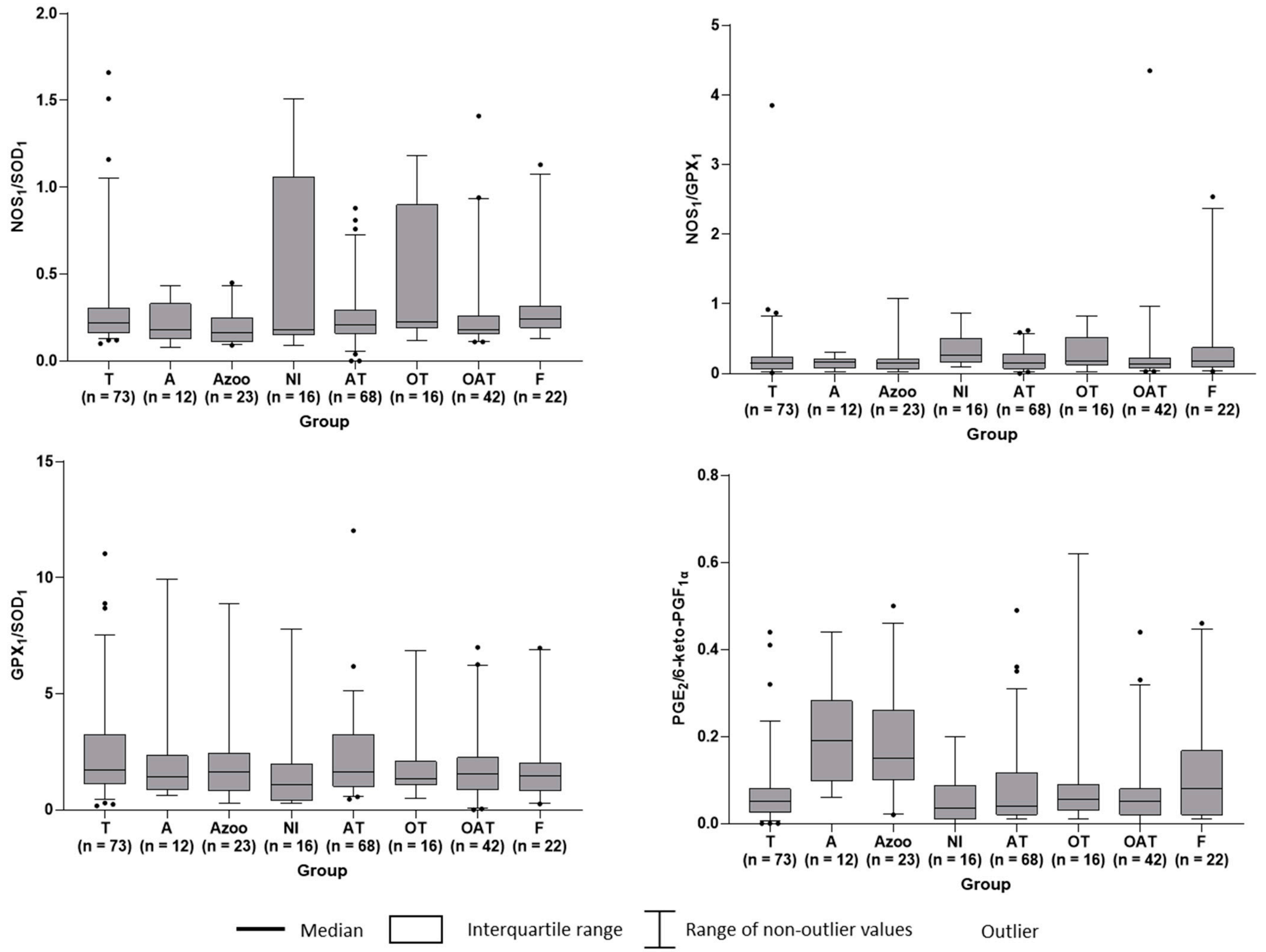

Figure S2. Comparison of ratios of examined parameters between groups of infertile men using box plots. 6-keto-PGF$_{1\alpha}$ – 6-keto-Prostaglandin F$_{1\alpha}$, A – Asthenozoospermic group, AT – Asthenoteratozoospermic group, Azoo – Azoospermic group, F – Fertile group, GPX$_1$ – Glutathione Peroxidase 1, NI – Normozoospermic infertile group, NOS$_1$ – Nitric Oxide Synthase 1, OAT – Oligoasthenoteratozoospermic group, OT – Oligoteratozoospermic group, PGE$_2$ – Prostaglandin E$_2$, SOD$_1$ – Superoxide Dismutase 1, T – Teratozoospermic group, n – number of participants.
